# An Elegant Method of One‐Pot Ligation‐Desulfurization for High‐Yielding Chemical Protein Synthesis

**DOI:** 10.1002/advs.202510194

**Published:** 2025-08-25

**Authors:** Vishal Malik, Abhisek Kar, Anandh Muthiah Venkatachalam, Kalyaneswar Mandal

**Affiliations:** ^1^ Tata Institute of Fundamental Research Hyderabad 36/p Gopanpally Hyderabad Telangana 500046 India

**Keywords:** native chemical ligation, chemical protein synthesis, one‐pot, desulfurization, solid phase peptide synthesis

## Abstract

While native chemical ligation (NCL), combined with radical‐mediated desulfurization, has enabled chemical syntheses of a vast array of proteins, the presence of aryl thiols as catalysts in the ligation mixture prevents performing both ligation and desulfurization reactions in one‐pot, as aryl thiols are effective radical scavengers. Existing one‐pot ligation and desulfurization approaches are not ideal as they rely on the use of inefficient alkyl thiols as NCL catalysts. Here an extremely efficient and impressively straightforward method is presented that utilizes bromoacetamide, in conjunction with N‐acetyl cysteine, for selective quenching of arylthiol following NCL, enabling ligation and desulfurization in one‐pot without any intermediate purification step. The reagent combination facilitates one‐pot reactions by selectively capping aryl thiols in the presence of peptidic cysteine residue, leveraging the increased nucleophilicity of aryl thiols over alkyl thiols. N‐acetyl cysteine additionally functions as the alkyl thiol additive for the desulfurization reaction. It is demonstrated the utility of this methodology by synthesizing three different proteins: ubiquitin, collagen, and barstar A. The strategy substantially enhances the efficiency of chemical protein synthesis, especially for cysteine‐free proteins. Its operational simplicity and broad applicability make this one‐pot ligation‐desulfurization protocol promising for widespread adoption in synthesizing therapeutic proteins and related biomolecules.

## Graphical abstract

A one‐pot native chemical ligation and desulfurization method has been developed for efficient chemical protein synthesis. This unique approach uses bromoacetamide and N‐acetyl cysteine to selectively quench arylthiol post native chemical ligation. The operational simplicity of the method enables high yield and broad applicability in synthesizing therapeutic proteins and other biologically relevant macromolecules.

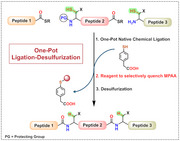

## 1 Introduction

Chemical protein synthesis has emerged as a powerful tool for the production of homogeneous proteins.^[^ ^1^ ^]^ The combination of two prime technologies, solid phase peptide synthesis (SPPS)^[^ ^2^, ^3^ ^]^ and native chemical ligation (NCL),^[^ ^4^ ^]^ has revolutionized the field of total chemical protein synthesis. These methods enable the precise construction of given protein sequences, which in turn facilitate the study of the structure and function of the protein of interest and accelerate the development of novel protein‐based therapeutic agents. The NCL starts with a chemoselective reaction between a C‐terminal peptide thioester and an N‐terminal cysteinyl peptide, forming a reversible thioester‐linked intermediate. Subsequently, an irreversible S‐to‐N acyl transfer occurs, leading to the formation of a native amide bond. For an efficient thiol‐thioester exchange reaction, an exogenous thiol catalyst is often necessary. Johnson and Kent have demonstrated that aryl thiols are among the most effective thiol catalysts for NCL.^[^ ^5^ ^]^ In particular, 4‐mercaptophenylacetic acid (MPAA) has been identified as a highly efficient catalyst, possessing balanced nucleophilicity and leaving group ability to facilitate NCL reaction.^[^ ^5^ ^]^ Consequently, MPAA is widely employed as an exogenous thiol catalyst for native chemical ligation.

Although native chemical ligation at cysteine residue enabled the synthesis of a variety of proteins, the requirement for a cysteine residue, the least abundant amino acid in natural proteins,^[^ ^6^ ^]^ at the *N*‐terminus of one of the reacting peptide segments however poses a significant challenge in the chemical synthesis of proteins. To address this issue, cysteine ​​has been used as a natural surrogate for alanine residues; and once the peptide bond is formed via NCL, the cysteine ​​is converted back to alanine via desulfurization. The desulfurization is achieved using either metal‐mediated protocols,^[^ ^7^, ^8^, ^9^ ^]^ or the non‐metal‐based methods,^[^ ^10^, ^11^, ^12^, ^13^, ^14^, ^15^, ^16^, ^17^, ^18^, ^19^, ^20^, ^21^ ^]^ including widely used radical initiator VA‐044^[^ ^19^, ^20^, ^21^ ^]^ in combination with the water‐soluble Tris‐carboxyethylphosphine hydrochloride (TCEP). Recent developments in protein desulfurization methods extend beyond cysteine and include unnatural β‐, γ‐, and δ‐thio‐derivative of amino acids, which upon desulfurization can regenerate the corresponding native amino acid other than alanine.^[^ ^1^, ^21^ ^]^ Advanced ligation methods, such as one‐pot multi‐segment NCL,^[^ ^22^, ^23^, ^24^, ^25^, ^26^, ^27^, ^28^, ^29^, ^30^ ^]^ followed by robust desulfurization protocols, have already been used to synthesize a wide spectrum of protein targets.^[^ ^23^, ^26^, ^30^ ^]^

Although the NCL followed by desulfurization offers a promising solution for producing larger polypeptides without cysteine ​​at the ligation sites, the ligation‐desulfurization cannot be performed in one‐pot when MPAA is present in the NCL reaction mixture, as aryl thiols are highly effective radical quenchers.^[^ ^31^ ^]^ Consequently, performing a desulfurization reaction after the MPAA‐mediated NCL reaction necessitates an additional purification step, which is a tedious, low‐yielding, and inefficient process. To obviate such a situation, less reactive, difficult to handle, and typically malodorous alkyl thiols, such as 2,2,2‐trifluoroethanethiol (TFET), methyl thioglycolate (MTG), sodium 2‐mercaptoethanesulfonate (MESNa) and thiocholine, are used as thiol catalysts for one‐pot ligation and desulfurization reaction.^[^ ^32^, ^33^, ^34^, ^35^, ^36^, ^37^ ^]^ Furthermore, a large excess of imidazole has also been utilized as a catalyst for NCL, enabling a one‐pot NCL‐desulfurization strategy. However, the study of one‐pot ligation‐desulfurization, especially in the presence of aryl thiols as catalysts, is relatively underexplored. To the best of our knowledge, only two approaches have been utilized to enable ligation‐desulfurization in one‐pot in the presence of MPAA. The first approach involves the use of preformed MPAA thioesters.^[^ ^8^, ^31^ ^]^ The second approach involves the functionalization of MPAA/arylthiol with a hydrazine moiety, which was then captured by a chemically synthesized aldehyde resin via an oxime formation to enable desulfurization.^[^ ^31^ ^]^ Neverthless, both approaches require additional effort, either in the synthesis and purification of the preformed MPAA thioester that is highly susceptible to hydrolysis, or in the functionalization of the MPAA thiol and the preparation of the aldehyde‐functionalized resin.

To overcome these limitations, herein we present an innovative and highly efficient one‐pot ligation‐desulfurization strategy in the presence of an aryl thiol catalyst. We used a reagent combination, consisting of inexpensive bromoacetamide and N‐acetyl cysteine, to selectively quench MPAA following NCL, forming a thioether within 5 min. This process took advantage of the higher nucleophilicity of aryl thiol compared to the peptidic cysteine. N‐acetyl cysteine also served as an alkyl thiol additive for the efficient desulfurization reaction. The quantitative quenching of MPAA present in the reaction mixture enabled the subsequent desulfurization reaction in one‐pot, eliminating the need for any intermediate purification step. We demonstrated the general applicability of this method by total chemical synthesis of proteins that do not contain cysteine ​​residues. Thus, in terms of both conceptual and technological advances, this work expands the scope of one‐pot methods for efficient chemical protein synthesis.

## 2 Results and Discussion

**Figure 1:**
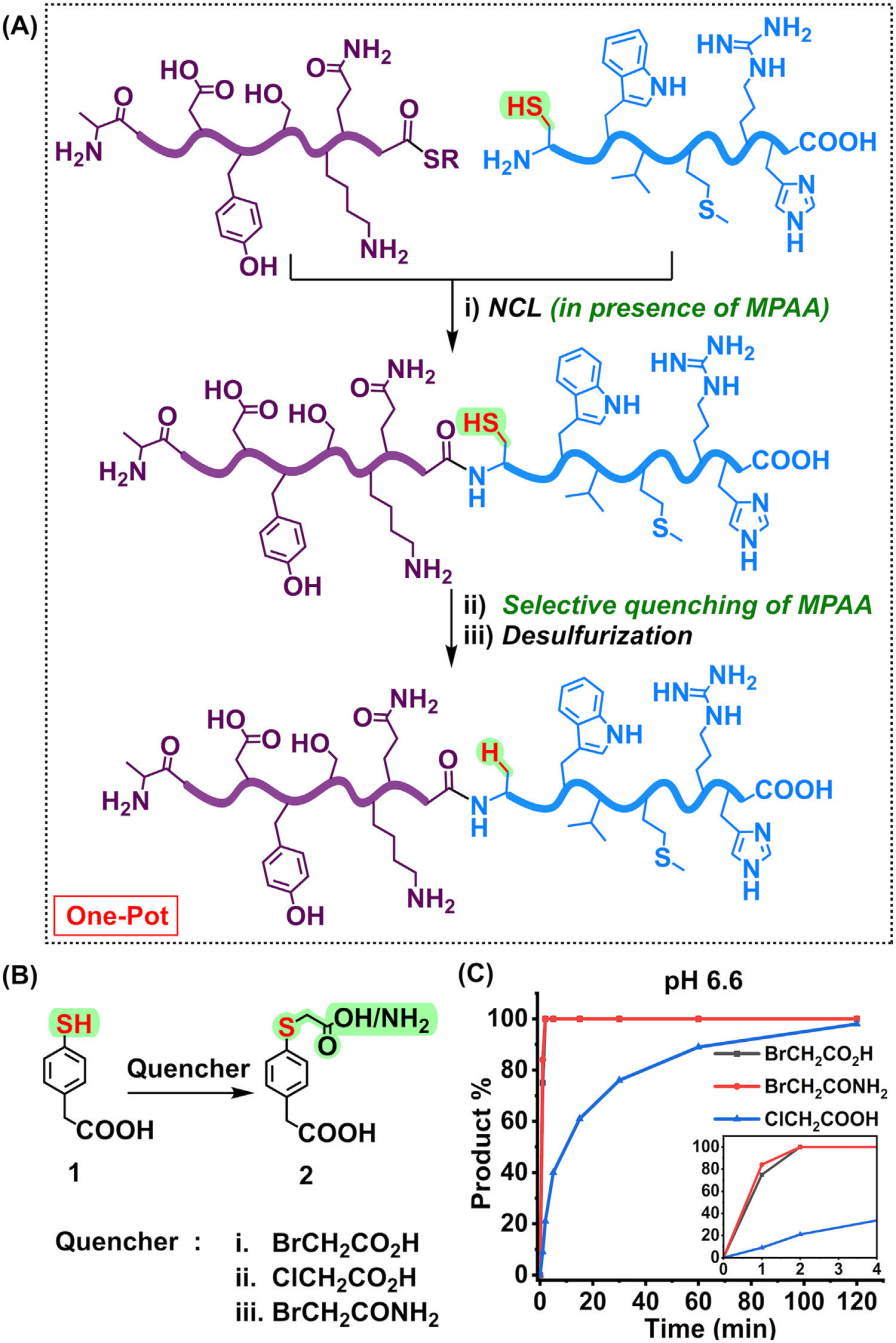
A) A general schematic of one‐pot ligation‐desulfurization strategy. B) Schematic of the reaction of MPAA with bromoacetic acid, bromoacetamide, and chloroacetic acid. C) Kinetic study of the reaction of 20 mm MPAA with 25 mm bromoacetic acid (black), bromoacetamide (red), and chloroacetic acid (blue) in the buffer (6 m Gu.HCl, 0.2 m phosphate, 20 mm TCEP) at pH 6.6.

For one‐pot NCL‐desulfurization, a crucial requirement was to find an optimal reagent that can selectively and rapidly block the aryl thiol catalyst in the presence of alkyl thiols (cysteines) within the peptide. To achieve this, we took advantage of the lower pKa value of aryl thiols (e.g., the pKa of MPAA is ≈6.6)^[^ ^5^ ^]^ than alkyl thiols (pKa of the internal cysteine of a peptide is ≈8.3), which leads to aryl thiols' higher nucleophilicity even in mildly acidic environments. First, we opted for two molecules, bromoacetic acid and chloroacetic acid, which tend to alkylate the thiol moieties and do not react with functional side‐chains of other amino acids below pH 7.0, unless used in excess. Our initial experiments with MPAA in the ligation buffer (6 m guanidine hydrochloride (Gu.HCl), 0.2 m phosphate, 20 mm TCEP) revealed that both reagents react with MPAA‐thiol at pH 3.0 and above and the reaction rate increases with increasing pH of the reaction mixture. In the presence of bromoacetic acid, we observed quantitative quenching of MPAA at pH 5.0 within 15 min. The reaction time was reduced to less than 5 min when the pH was adjusted nearly to the ligation pH (6.6). In contrast, the presence of chloroacetic acid resulted in a significantly slower rate of quenching. At pH 5.0, complete alkylation of MPAA took ≈15 h, whereas increasing the pH to 6.6 accelerated the reaction and shortened the completion time to ≈2 h (**Figure** **1**). Hence, we considered bromoacetic acid as a superior thiol quencher over chloroacetic acid due to its greater reactivity with aryl thiols. However, being highly acidic, the addition of bromoacetic acid significantly lowered the pH of the reaction mixture, making it difficult to maintain a stable pH during the course of the reaction. Also, the hygroscopic nature of bromoacetic acid made the precise weighing of the reagent challenging. Therefore, we set out to use the non‐hygroscopic and pH‐neutral amide form, that is bromoacetamide instead, as it does not significantly alter the pH of the buffer, is easier to weigh out accurately, and reacts just as rapidly as bromoacetic acid with the aryl thiols (Figure 1B–D).

Next, to assess the specificity of bromoacetamide to MPAA thiol over peptidic cysteine, we carried out three distinct reactions using 1 mm model peptide *Ac*‐CFRAL‐*^α^COOH* (**1**) in the presence of 20 mm MPAA with different bromoacetamide concentrations (20, 30, and 40 mm) in the ligation buffer (6 m Gu.HCl, 0.2 m phosphate, 20 mm TCEP, pH 6.8). We observed near quantitative alkylation of MPAA at just 1 equivalent (20 mm) bromoacetamide, leaving only a trace amount of unreacted MPAA and ≈8% of the alkylated cysteine ​​of the peptide as the byproduct. As expected, a higher percentage of alkylated‐cysteinyl peptide byproduct was formed when an increased amount of bromoacetamide was used, highlighting the importance of optimizing the reaction condition to minimize the side product formation (Figure S7, Supporting Information).

To minimize the formation of the undesired alkylation of the internal cysteine of the peptide, we explored the addition of exogenous alkyl thiol to the reaction mixture prior to the addition of bromoacetamide, keeping in mind that the added exogenous alkyl thiol will also play the role of thiol additive during subsequent desulfurization step. To test this approach, we maintained the above reaction conditions with 20 mm bromoacetamide and evaluated the reactivity of five alkyl thiols, MESNa, cysteine, N‐acetyl cysteine, sodium 3‐mercapto‐1‐propanesulfonate and cysteamine, at a 100 mm concentration to compete with the internal cysteine ​​thiol of the peptide. Our results indicated that cysteamine and MESNa did not significantly reduce the unwanted alkylation of internal cysteine byproducts, whereas N‐acetyl cysteine, having similar pKa as peptidic cysteine, ​​reduced the byproduct formation from 8% to <2.5% (Figure S8, Supporting Information).

To further determine the selectivity of MPAA for bromoacetamide over other reactive amino acids, we synthesized another model peptide, STFTKSPC ‐*^α^CONH_2_* (**2**), which contains amino acids with nucleophilic side chains, ─NH_2_ or ─OH. The reaction mixture was comprised of 1 mm peptide **2**, 20 mm MPAA, and 20 mm TCEP in the ligation buffer (6 m Gu.HCl, 0.2 m phosphate), to which 100 mm N‐acetyl cysteine ​​was added, followed by 20 mm bromoacetamide. Our results showed that the MPAA is selectively quenched at pH 6.6, with a negligible amount of peptide being alkylated, which correlates with the outcomes of the reaction with model peptide **1** and corroborates that only the cysteine ​​residue results in minimal byproduct formation (<2.5%) (Figure S9, Supporting Information).

**Figure 2:**
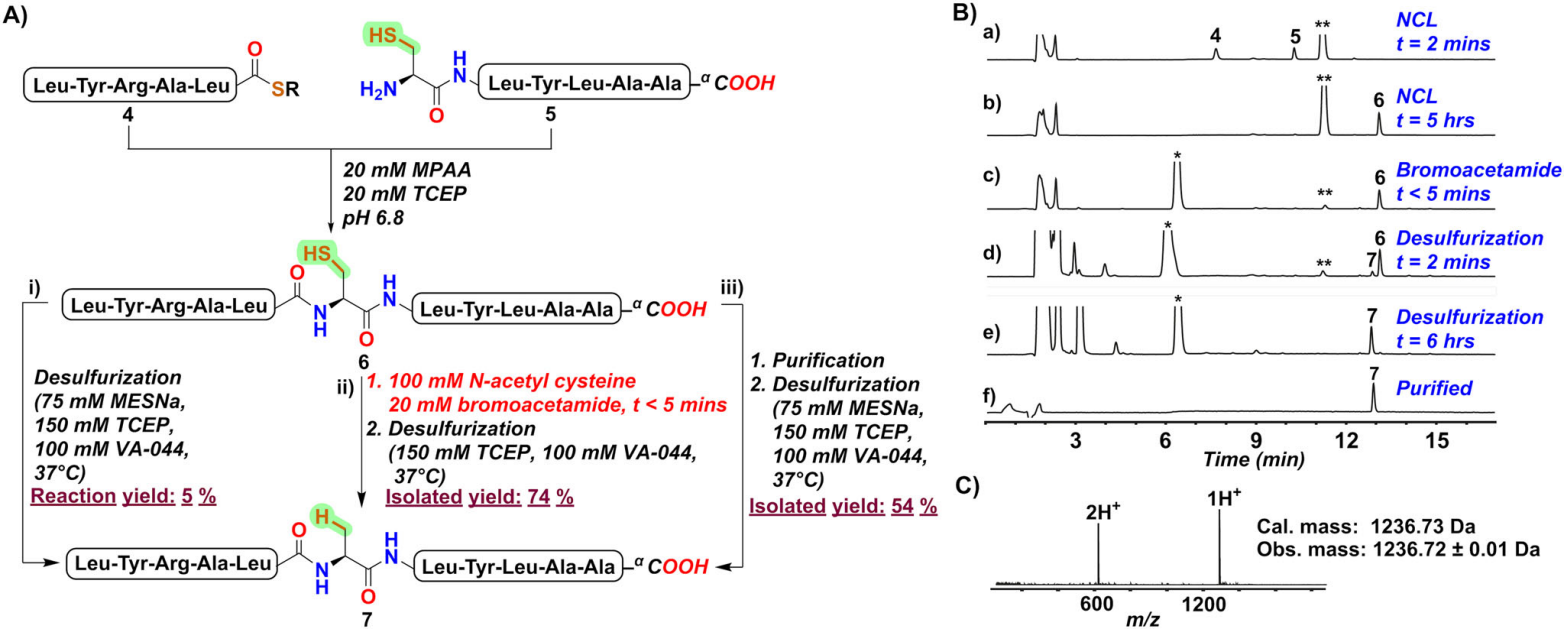
A) A strategy for the synthesis of polypeptide **7** by three different routes (i) NCL followed by direct desulfurization (ii) NCL followed by quenching of MPAA and then one‐pot desulfurization (iii) NCL followed by purification and desulfurization. B) Analytical HPLC chromatogram (λ = 214 nm) for all the steps of the synthesis of polypeptide **7** as described in A(ii). C) The ESI‐MS data of the purified polypeptide **7**. The ^**^ indicates MPAA and ^*^ indicates alkylated MPAA; *R = −CH_2_CH_2_SO_3_Na*, *MPAA* = *HS‐C_6_H_4_CH_2_COOH*.

Finally, to validate the one‐pot ligation‐desulfurization with the optimized arylthiol quenching strategy, we synthesized two model peptides LYRAL‐*^α^CONHNH_2_* (**3**) and CLYLAA‐*^α^COOH* (**5**). Peptide **3** was converted to thioester (LYRAL‐*^α^COSR*, R = ‐CH_2_CH_2_SO_3_Na, **4**) by NaNO_2_‐mediated oxidation followed by thiolysis using MESNa under aqueous conditions.^[^ ^38^ ^]^ The two peptides **4** and **5** were ligated and subsequently desulfurized in the same reaction mixture without any purification. We performed three different reactions to evaluate the effectiveness of our one‐pot ligation‐desulfurization approach: i) ligation of both peptides **4** and **5** in the presence of MPAA, followed by direct desulfurization using standard condition without capping or purification; ii) ligation followed by capping of MPAA with bromoacetamide in the presence of N‐acetyl cysteine ​​and subsequent one‐pot desulfurization; iii) ligation followed by reverse phase HPLC purification to remove the MPAA and subsequent desulfurization with purified ligated product (**Figure** **2**). For the first reaction, reagents for the desulfurization (MESNa (75 mm), TCEP (150 mm) and VA‐044 (100 mm)) were introduced in the crude reaction mixture post‐ligation at 37 °C without quenching the MPAA, which resulted in only 5% conversion to the desulfurized product even after 12 h (Figure S10, Supporting Information) as expected. In the second reaction, ligation was completed within 5 h at pH 6.8. To this solution, 100 mm N‐acetyl cysteine was added, followed by 20 mm bromoacetamide at the same pH, effectively quenching the MPAA within 5 min. The addition of TCEP (150 mm) and VA‐044 (100 mm) in the same reaction mixture enabled radical‐mediated desulfurization at 37 °C within 6 h, resulting in 74% pure isolated product **7** (Figure 2,B). In the third reaction, ligated product **6** was purified. This was followed by a standard desulfurization process which was completed in 6 h and resulted in an overall isolated yield of 54% of the product **7** (Figure S12, Supporting Information). This demonstrates the effectiveness of the bromoacetamide reagent in selectively capping the MPAA thiol in the presence of N‐acetyl cysteine. This approach not only saved the time required for purification and lyophilization but also resulted in significantly high overall yield of the end product.

In order to demonstrate the general applicability of this approach to thiol‐containing other amino acid surrogates, we synthesized a model peptide containing D‐penicillamine, a surrogate for valine^[^ ^20^ ^]^ with the sequence A(**Pen**)GFRAL‐*^α^NH_2_*, and desulfurized in one‐pot. To mimic the ligation conditions, we added 20 mm MPAA and 20 mm TCEP along with 1 mm penicillamine‐containing peptide in the ligation buffer (6 m Gu.HCl, 0.2 m PB, pH 6.8) followed by the addition of 100 mm N‐acetyl cysteine. To this solution, 20 mm bromoacetamide was added resulting in the selective quenching of MPAA within 5 min. Subsequently, 150 mm TCEP and 100 mm VA‐044 were added to the same reaction mixture adjusting the pH to 7.0, and incubated at 37 °C, which resulted in the complete desulfurization of the penicillamine‐thiol group within 8 h (Figure S13, Supporting Information), confirming that this method can very well be used for the one‐pot desulfurization of thiol‐containing other native amino acid surrogates as well.

To demonstrate the adaptability of our approach with the recently published fascinating ultrasound‐induced protein desulfurization (USID) protocol by Liu group^[^ ^8^ ^]^ that uses ultrasonic wave energy along with TiO_2_ nanoparticles to effectively desulfurize the proteins, we examined USID of the model peptide Ac‐CFRAL using our one‐pot strategy. Mechanistically, USID proceeds via a radical‐mediated pathway promoted by low‐frequency ultrasonication and inhibited by the presence of aryl thiol catalyst, MPAA. To achieve one‐pot desulfurization, Liu group used pre‐formed peptide‐MPAA thioester. To evaluate the compatibility of our strategy with USID in presence of MPAA, we used 1 mm *Ac*‐CFRAL‐^α^ *COOH* (**1**) peptide in the presence of 20 mm MPAA in the ligation buffer (6 m Gu.HCl, 0.2 m phosphate, 20 mm TCEP) along with 100 mm N‐acetyl cysteine. MPAA thiol was then quenched using 20 mm bromoacetamide within 5 min followed by the addition of 200 mm TCEP and TiO_2_ (0.5 mg mL^−1^) nanoparticles. We incubated the reaction mixture in an ultrasonic bath at 37 °C. The peptide **1** was completely desulfurized within 7 h as shown in the Figure S14 (Supporting Information).

After a successful demonstration of the protocol with thiol‐containing various model peptides, we sought to demonstrate the general utility and effectiveness of our method by chemically synthesizing three different cysteine‐free proteins of amenable sizes: ubiquitin, a collagen analogue, and barstar A.

**Figure 3:**
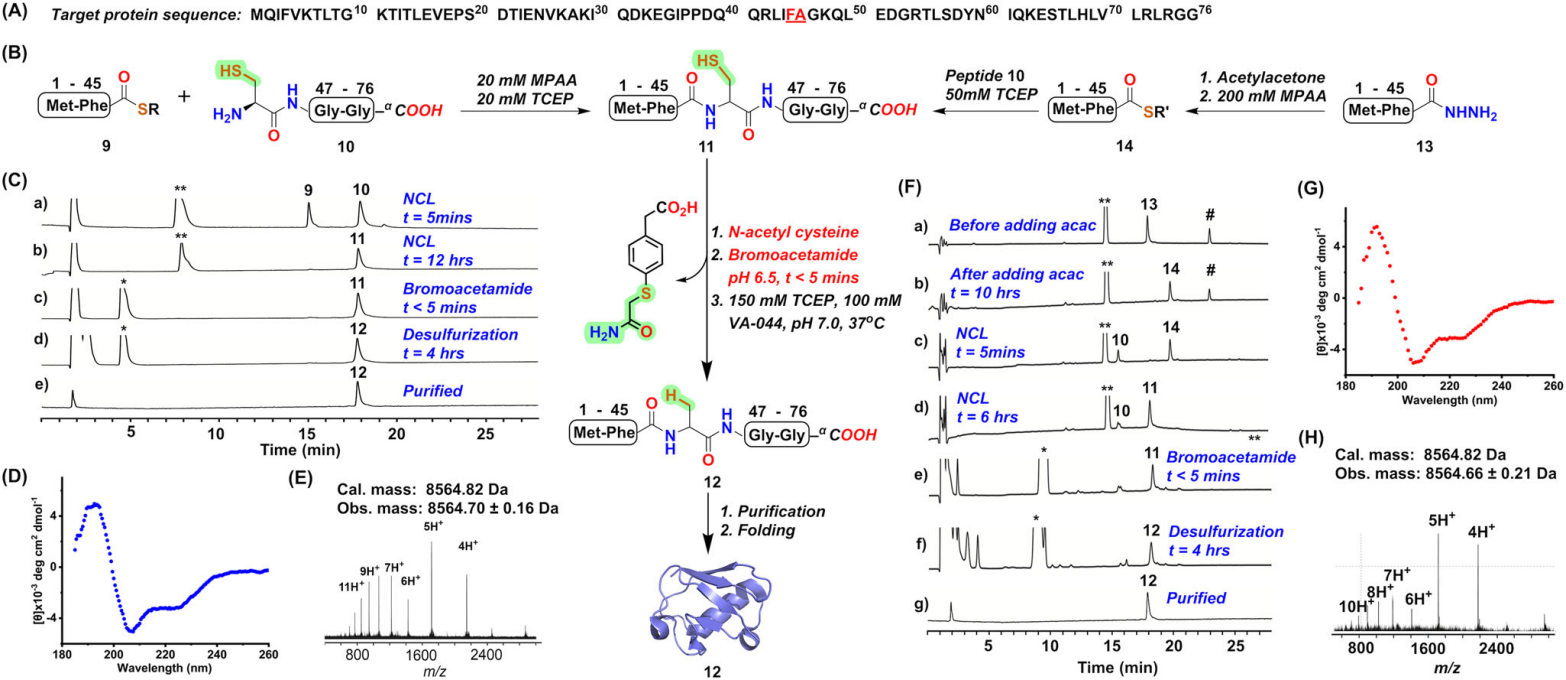
Total chemical synthesis of the ubiquitin protein. A) The amino acid sequence of ubiquitin. B) Synthetic strategy for the full‐length ubiquitin polypeptide **12** in two different routes. From left, NCL between peptides **9** and **10** in presence of MPAA yielded the ligated polypeptide **11**. From the right, activation of peptide hydrazide **13** by acetylacetone and formation of the corresponding MPAA thioester followed by NCL after the addition of peptide **10** to furnish the ligated product **11**. In the next step, MPAA is quenched by bromoacetamide in the presence of N‐acetyl cysteine followed by desulfurization leading to the desired ubiquitin full‐length polypeptide **12**. The cartoon representation of **17** was taken from PDB ID 1YIW. C) Analytical HPLC chromatogram (λ = 214 nm) for all the steps of the synthesis of polypeptide **12** as described in B (left to right). D) The far‐UV CD spectrum of the purified ubiquitin **12** following the route described in B (left to right), was obtained by measuring the ellipticity of the sample (an average of three repeats shown in blue). E) The ESI‐MS data of the purified ubiquitin protein **12** (average isotope), as described in B (left to right). F) Analytical HPLC chromatogram (λ = 214 nm) for all the steps of the synthesis of polypeptide **12** as described in B (right to left). G) The far‐UV CD spectrum was obtained by measuring the ellipticity of the sample (an average of three repeats shown in red). H) The ESI‐MS data of the purified ubiquitin protein **12** (average isotope), as described in B (right to left). The ^**^ indicates MPAA, ^*^ indicates alkylated MPAA, and **#** represents MPAA disulfide. *R = −CH_2_CH_2_SO_3_Na*; *R’ = −C_6_H_4_CH_2_COOH; MPAA* = *HS‐C_6_H_4_CH_2_COOH*.

*Total chemical synthesis of ubiquitin*. Ubiquitin is a unique protein found in all eukaryotic cells that serves as a molecular tag crucial for cellular regulation. With the help of covalent attachment to the target proteins, it orchestrates diverse cellular functions, such as proteasomal degradation, the regulation of the chromatin structure, and protein localization.^[^ ^39^, ^40^ ^]^ The chemical synthesis of ubiquitin has enabled researchers to investigate its biochemical and biophysical properties in greater detail.^[^ ^41^, ^42^ ^]^ We synthesized ubiquitin (Ub) from two segments, Met^1‐^Phe^45^‐Dbz‐*^α^CONH_2_* (**8**) and Cys^46‐^Gly^76^‐*^α^COOH* (**10**). Dbz stands for 3,4‐diaminobenzoic acid.^[^ ^43^, ^44^ ^]^ Peptide **8** was converted to the thioester (Met^1‐^Phe^45^‐*^α^COSR*, R = ‐CH_2_CH_2_SO_3_Na, **9**) by NaNO_2_‐mediated activation of the peptide o‐aminoanilide followed by thiolysis using MESNa in aqueous conditions.^[^ ^45^ ^]^ To enable NCL, Ala^46^ was replaced with Cys^46^ in peptide **10**. Ligation between peptides **9** and **10** in presence of MPAA was achieved within 12 h. Subsequently, the MPAA was capped with bromoacetamide within 5 min in the presence of N‐acetyl cysteine ​​and finally desulfurized within 4 h at 37 °C (**Figure** **3C**). The final product was then purified using reverse phase chromatography with a high isolated yield (74%), demonstrating the efficiency of this method as it not only saved time but also provided a significant yield as no purification was required for the intermediate steps.

To highlight the robustness of this method, we also explored alternate ways of generating peptide thioester during the synthesis of ubiquitin. Dawson et al. reported a novel thioesterification strategy from peptide hydrazide utilizing Knorr pyrazole synthesis method.^[^ ^46^ ^]^ Peptide thioesterification using this method enables sequential ligations under mild and stoichiometric condition. The generation of peptide thioester from hydrazide using the Knorr pyrazole synthesis method is compatible with N‐terminal thiazolidine, unlike other NaNO_2_ mediated Dbz or hydrazide conversion.^[^ ^38^, ^45^ ^]^ In this approach, stoichiometric acetylacetone reacts with the C‐terminal hydrazide to form N‐acylpyrazole. This intermediate undergoes in‐situ thioesterification with an external aryl thiol, such as MPAA, under acidic aqueous conditions around pH 3.0. We used this method to synthesize ubiquitin from the initial hydrazide segment Met^1^‐Phe^45^‐*^α^NHNH_2_* (**13**). To initiate thioesterification, MPAA (200 mm) and acetylacetone (2.5 equivalents of peptide **13**) were added with peptide **13** dissolved in 6 m Gu.HCl. The suspension was stirred at room temperature for 10 h. Peptide **10** dissolved in buffer (6 m Gu.HCl, 0.2 m phosphate, pH 9.0) containing TCEP (50 mm) was then introduced into the solution and final pH was adjusted to 6.9. After 6 h, HPLC‐MS analysis confirmed the formation of polypeptide **11**. The reaction mixture was diluted with the buffer (6 m Gu.HCl, 0.2 m phosphate, pH 7.0) to achieve a final concentration of peptide to 1 mm and MPAA to 40 mm. To this solution, 100 mm N‐acetyl cysteine ​​was added, followed by the addition of bromoacetamide (40 mm) at pH 6.5 to quench the MPAA present in the reaction mixture. The addition of TCEP (150 mm) and VA‐044 (100 mm) resulted in the desulfurization of polypeptide **11** within 4 h at 37 °C (Figure 3F). The final product **12** was isolated in a very high yield (67%). The circular dichroism (Figure 3D,G) and the ESI‐MS data (Figure 3E,H) of the folded ubiquitin protein obtained via both routes revealed the characteristic signature of the secondary structural elements and accurate molecular mass demonstrating the versatility of this method.

**Figure 4:**
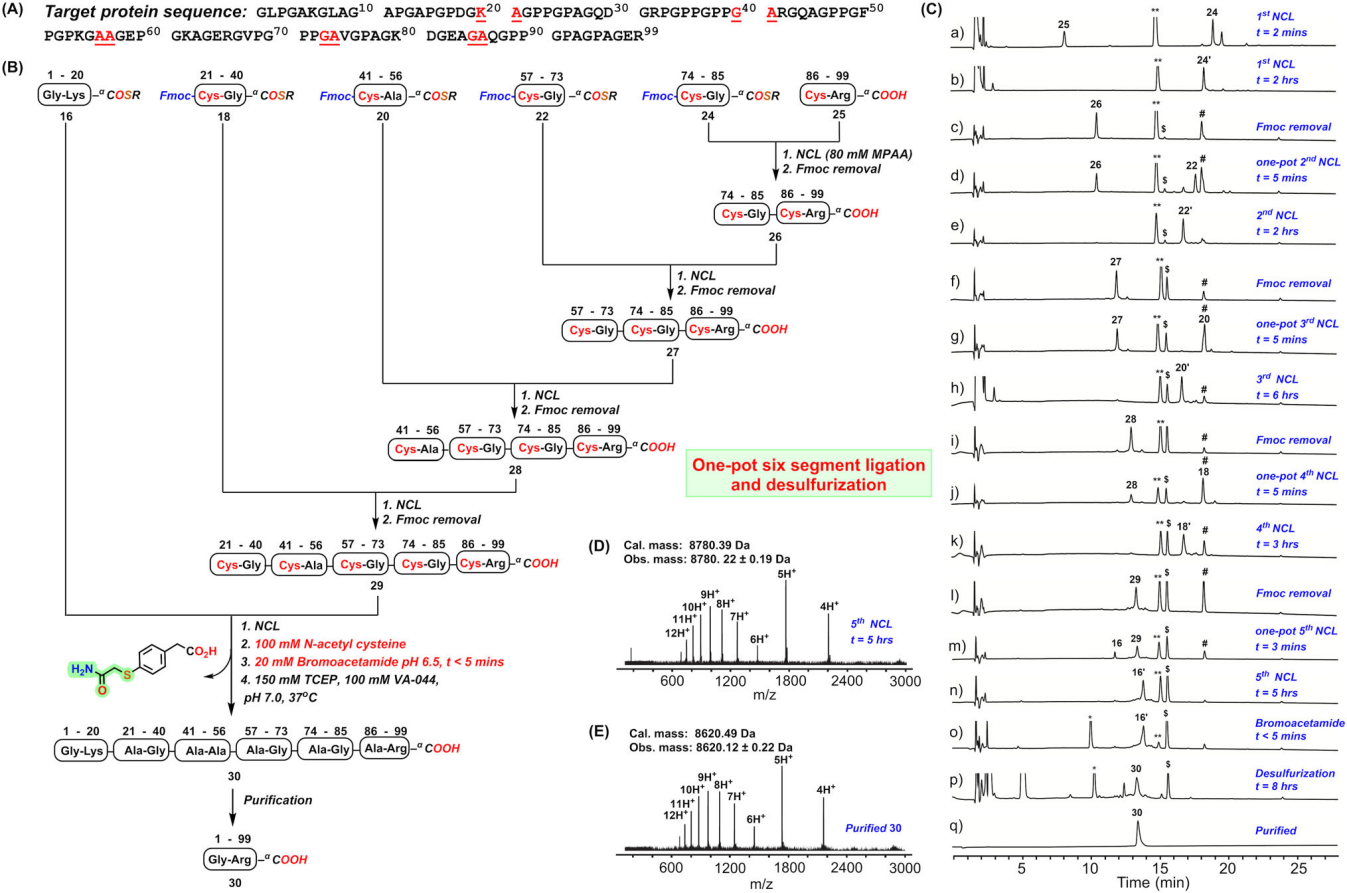
Total chemical synthesis of collagen protein. A) The amino acid sequence of collagen protein synthesized as reported^[^ ^48^ ^]^ B) Strategy for the synthesis of the full‐length collagen polypeptide **30** from six peptide segments in one‐pot in presence of MPAA. C) Analytical HPLC chromatogram (λ = 214 nm) for all the steps of the synthesis of polypeptide **30** as described in B. D) The ESI‐MS data (average isotope) of the full‐length polypeptide **16′** after six‐segment one‐pot ligations. E) The ESI‐MS data of the purified collagen protein **30**. The ^**^ indicates MPAA, ^*^ indicates alkylated MPAA, $ represents dibenzofulvene‐TCEP adduct, and **#** represents dibenzofulvene‐piperidine adduct. *R = −CH_2_CH_2_SO_3_Na*, *MPAA* = *HS‐C_6_H_4_CH_2_COOH*.

*Total chemical synthesis of collagen analogue*. Collagen is the most prevalent protein in all animals, which is essential for providing structural support to various tissues, including skin, bones, tendons, and cartilage.^[^ ^47^ ^]^ This fibrous, structural protein constitutes ≈75% of the dry weight of skin and is the predominant component of the extracellular matrix (ECM). A collagen analogue, where hydroxy‐proline is replaced by proline, has recently been used as a model protein for showcasing fast‐flow chemical protein synthesis.^[^ ^48^ ^]^ To demonstrate the feasibility of one‐pot multi‐segment NCL‐desulfurization reaction, we synthesized this 99‐residue collagen analogue from six segments: Gly^1^‐Lys^20^‐^α^ *COSR* (**16**), Cys^21^‐Gly^40^‐^α^ *COSR* (**18**), Cys^41^‐Ala^56^‐^α^ *COSR* (**20**), Cys^57^‐Gly^73^‐^α^ *COSR* (**22**), Cys^74^‐Gly^85^‐^α^ *COSR* (**24**), and Cys^86^‐Arg^99^‐^α^ *COOH* (**25**) (R = ‐CH_2_CH_2_SO_3_Na) in presence of MPAA followed by desulfurization in a one‐pot fashion (**Figure** **4**B). To ligate six segments, we used a one‐pot C‐to‐N multi‐segment ligation strategy using Fmoc‐group as a temporary protecting group of the N‐terminal cysteine of internal peptide segments.^[^ ^29^ ^]^ The initial ligation between peptides **24** and **25** was completed in 2 h, followed by piperidine (20% v/v) addition to attain a pH of 11.0 for the Fmoc‐group removal within 7 min. After completion of the Fmoc‐deprotection, the pH of the solution was immediately lowered using concentrated HCl. Solid TCEP (80 mm) was then added, and the pH of the reaction was adjusted to 6.8 for the second ligation. Subsequent segments were ligated sequentially by cycling the pH to 11 for Fmoc‐removal and back to 6.8 for the ligation, yielding the full‐length 99‐residue ligated polypeptide **16′**. After the final ligation was completed, 100 mm N‐acetyl cysteine ​​was added, followed by the addition of bromoacetamide to an equivalent amount of MPAA (Note: *This needs to be calculated based on the equivalence of MPAA added at the start of the ligation*). After 5 min, the addition of TCEP (150 mm), and *VA‐044* (100 mm) resulted in the desulfurization of the polypeptide **16′** within 8 h at 37° C. Product **30** was isolated in high yield (43%). The ligations and desulfurization were analyzed by HPLC‐MS, which clearly showed the formation of the full‐length collagen polypeptide **30**. To the best of our knowledge, this is the first demonstration of a one‐pot six‐segment ligation and desulfurization for the chemical synthesis of protein.

**Figure 5:**
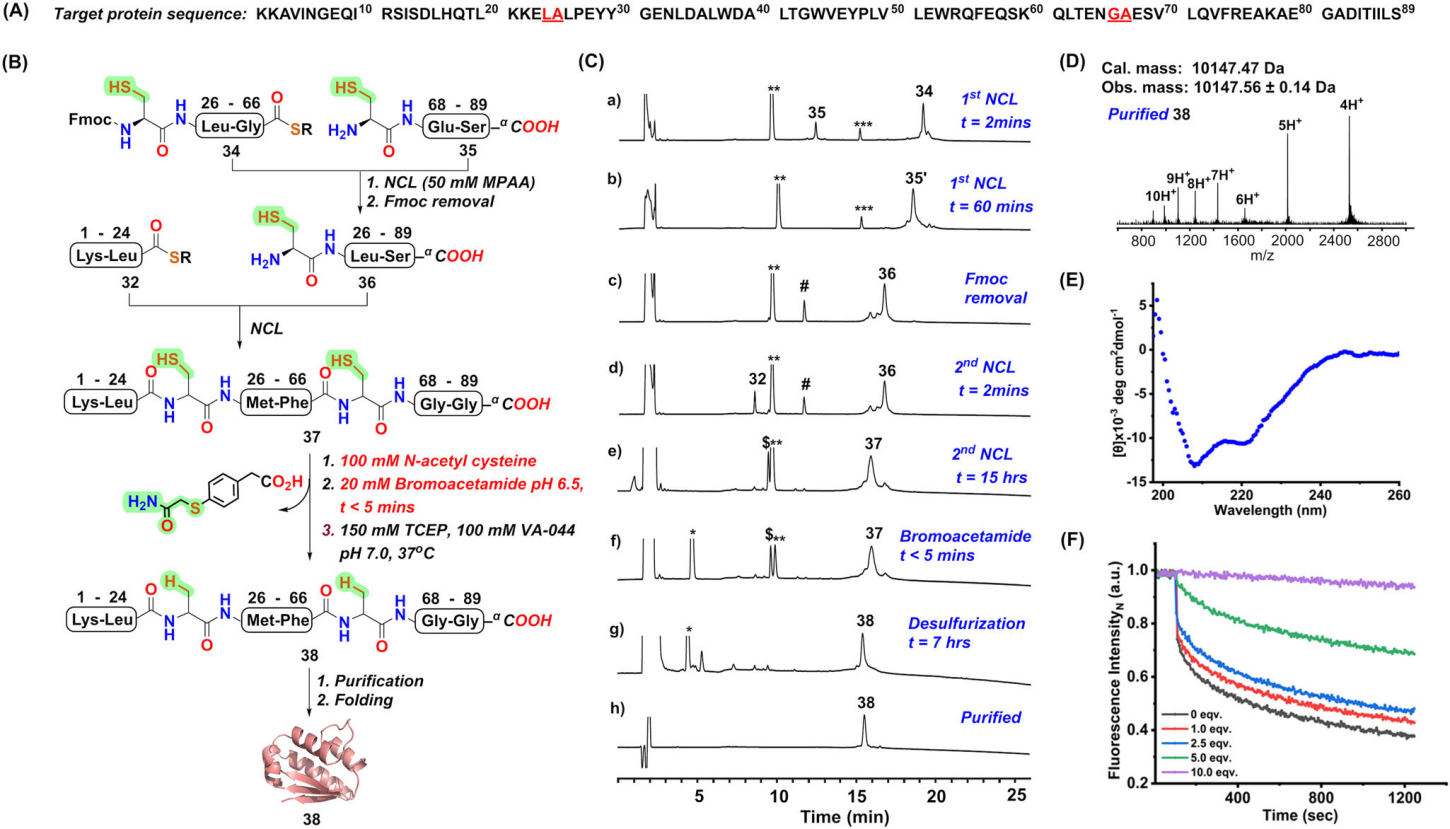
Total chemical synthesis of Barstar A. A) The amino acid sequence of Barstar A B) Strategy for the synthesis of the full‐length Barstar A polypeptide **38**. The cartoon representation of **38** was taken from PDB 2ZA4 C) Analytical HPLC chromatogram (λ = 214 nm) for all the steps of the synthesis of polypeptide **38** as described in B. D) The ESI‐MS data of the full‐length purified Barstar A polypeptide **38** (average isotope). E) The far‐UV CD spectrum of folded **38**, was obtained by measuring the ellipticity of the sample (an average of three repeats shown in blue) in 20 mm PB, 100 mm NaCl, pH 8.0 buffer at 25 °C temperature. F) Relationship between the changes in fluorescence intensity of the EtBr–RNA system with increasing concentrations (0.4, 1, 2.5, and 4 µm) of chemically synthesized Barstar A (0 to 10 eq) in the presence of 400 nm of Barnase. λ_ex_ = 520 nm. The ^**^ indicates MPAA; ^*^ indicates alkylated MPAA; $ represents dibenzofulvene‐TCEP adduct and **#** represents dibenzofulvene‐piperidine adduct. *R = −CH_2_CH_2_SO_3_Na*, *MPAA* = *HS‐C_6_H_4_CH_2_COOH*.

Finally, to further demonstrate the general utility of our method we synthesized a mutant variant of the barstar protein, named barstar A, which binds to the active site of the ribonuclease barnase and completely inhibits its activity.^[^ ^49^ ^]^ The active site of barnase contains positively charged residues that facilitate its interaction with RNA. In contrast, the active site of barstar A includes negatively charged residues that allow electrostatic attachment to barnase, forming a highly stable complex with strong affinity. The mutagenesis study revealed that the presence of a single disulfide bond is not essential for its inhibitory function.^[^ ^50^ ^]^ Barstar A, specifically the Cys40,82Ala mutant of barstar, was the first variant to be crystallized in complex with barnase, because it did not aggregate and therefore formed single crystals. The affinity of the mutated barstar A is reported with a dissociation constant on the order of tens of femtomolar.^[^ ^51^ ^]^ We divided this 89‐residue protein into three segments: Lys^1^‐Leu^24^‐^α^ *COSR* (**32**), *Fmoc*‐Cys^25^‐Gly^66^‐^α^ *COSR* (**34**), and Cys^67^‐Ser^89^‐^α^ *COOH* (**35**), with Ala^25^, and Ala^67^ mutated to cysteine to serve as ligation sites for the two ligations (**Figure** **5**A,B). Using a one‐pot C‐to‐N multi‐segment ligation strategy, we ligated the three segments in the presence of MPAA in a one‐pot fashion and then desulfurized them without any purification. The first ligation between **34** and **35** was completed within 1 h, which was followed by the removal of the Fmoc group in one‐pot. The subsequent ligation between **32** and **36** took 15 h. The remaining MPAA in the buffer was quenched by treatment with an equivalent amount of bromoacetamide in the presence of *N*‐acetyl cysteine, followed by desulfurization within 8 h. The final reverse phase purification gave pure barstar A (**38**) with an isolated overall yield of 35% (Figure 5C,D).

Circular dichroism (CD) spectrum revealed the presence of characteristic secondary structural elements in the folded barstar A protein, consistent with previously reported CD spectra of barstar A^[^ ^50^ ^]^ (Figure 5E). Furthermore, to demonstrate that the chemically synthesized barstar A is functionally active, we performed an inhibition assay of barstar A against its natural binder barnase, following the protocol that used the intercalating reagent ethidium bromide (EtBr) that binds with RNA and the change in fluorescence emission intensity of EtBr was monitored.^[^ ^52^ ^]^ Our results revealed that chemically synthesized barstar A effectively inhibits the ribonuclease activity of barnase. This is evinced by the increased fluorescence intensity of EtBr intercalated with RNA after preincubation of various equivalents (1.0, 2.5, 5.0, and 10.0) of barstar A with 400 nm barnase compared to the ribonuclease activity of 400 nm barnase alone (Figure 5F). Our results are consistent with previously published barstar protein activity data.^[^ ^48^ ^]^ This experiment confirmed the functional integrity of the chemically synthesized barstar A prepared by one‐pot multi‐segment ligation‐desulfurization protocol.

## 3 Conclusion

To integrate the two highly efficient reactions, NCL and desulfurization, into an operationally simple one‐pot protocol, we used a combination of commercially available bromoacetamide and N‐acetyl cysteine reagents for rapid and selective quenching of the radical scavenger arylthiol present in the ligation mixture. More importantly, the one‐pot NCL‐desulfurization protocol described here allowed us to use more reactive MPAA instead of less reactive, difficult‐to‐handle, and malodorous alkylthiols as catalyst for NCL reaction. We highlight the utility of bromoacetamide as a selective capping reagent for MPAA thiol, and N‐acetyl cysteine serving as an alkyl thiol additive, enabling one‐pot ligation‐desulfurization reactions on both model peptide systems having cysteine or penicillamine as well as in the assembly of multiple peptide fragments for access to functional proteins. Specifically, we used the capping reagent for the efficient one‐pot synthesis of three very different proteins: ubiquitin, collagen, and barstar A. Given the speed, efficiency, and simplicity of the protocol using a combination of N‐acetyl cysteine and bromoacetamide as a selective capping reagent for aryl thiols after ligation, we anticipate that it will find widespread application in the chemical synthesis of proteins that require desulfurization steps, thereby significantly increasing the efficiency of the processes. The selective capping of MPAA thiol goes beyond its application in one‐pot desulfurization. It can be used in various scenarios where the thiol group inhibits subsequent reactions, such as one‐pot Acm removal and one‐pot reaction of the cysteine ​​group after ligation with an alkylating reagent for peptide/protein bioconjugation.

## Conflict of Interest

The authors declare no conflict of interest.

## Supporting information



Supporting Information

## Data Availability

The data that support the findings of this study are available in the supplementary material of this article.
