# Supplementary material for: An Elegant Method of One‐Pot Ligation‐Desulfurization for High‐Yielding Chemical Protein Synthesis
Source: Adv Sci (Weinh). 2025 Aug 25;12(38):e10194. doi: 10.1002/advs.202510194 (PMC12520469; doi:10.1002/advs.202510194)
Supplement: Supplementary file 1 — Supporting Information [file ADVS-12-e10194-s001.pdf]

ADVANCED SCIENCE Open Access

Supporting Information

for *Adv. Sci.*, DOI 10.1002/advs.202510194

An Elegant Method of One-Pot Ligation-Desulfurization for High-Yielding Chemical Protein Synthesis

*Vishal Malik, Abhisek Kar, Anandh Muthiah Venkatachalam and Kalyaneswar Mandal**

An Elegant Method of One-pot Ligation-desulfurization for High-yielding Chemical Protein Synthesis

Vishal Malik, Abhisek Kar, Anandh Muthiah Venkatachalam and Kalyaneswar Mandal*

V. Malik, A. Kar, A M. Venkatachalam, Prof. Dr. K. Mandal
Tata Institute of Fundamental Research Hyderabad
36/p Gopanpally, Hyderabad, Telangana – 500046, India
E-mail: kmandal@tifrh.res.in

1. General Methods 3
1.1. Reagents 3
1.2. Reverse-phase HPLC and LC-MS analysis..3
1.3. A general protocol for machine-assisted Fmoc-SPPS..3
1.4. A general protocol for manual Fmoc-SPPS 4
1.5. Circular dichroism..4
2. Model peptide synthesis 4
2.1. Synthesis of *Ac*-Cys-Phe-Arg-Ala-Leu-$^{\alpha}$*COOH* (1) 4
2.2. Synthesis of model peptide Ala-Pen-Gly-Phe-Arg-Ala-Leu-$^{\alpha}CONH_2$ (1α)..5
2.3. Synthesis of model peptide Ser-Thr-Phe-Thr-Lys-Ser-Pro-Cys-$^{\alpha}CONH_2$ (2) 5
2.4. Synthesis of model peptide Leu-Tyr-Arg-Ala-Leu-$^{\alpha}$*COSR* (4)..6
2.5. Synthesis of model peptide Cys-Leu-Tyr-Leu-Ala-Ala-$^{\alpha}$*COOH* (5) 6
3. Optimization of the reactions of MPAA with alkylating reagents..7
3.1. Rate of the reaction between MPAA and alkylating reagents at pH 5 and 6.6 7
3.2. Evaluating the specificity of bromoacetamide towards MPAA in the presence of model peptide 1..8
3.3. Effect of alkyl thiol additives on the alkylation (by-product) of peptide 1 8
3.4. Reaction of MPAA with bromoacetamide in the presence of N-acetyl cysteine and model peptide 2 9
4. Ligation between two model peptides 4 and 5 followed by desulfurization under different conditions 10
4.1. Ligation between two model peptides 4 and 5 followed by direct desulfurization..10
4.2. Ligation between two model peptides 4 and 5 followed by quenching of MPAA and desulfurization..10
4.3. Ligation between two model peptides 4 and 5 followed by purification and desulfurization 11
5. Desulfurization of Ala-Pen-Gly-Phe-Arg-Ala-Leu-$^{\alpha}CONH_2$ (1α) in the presence of MPAA 12
6. One-pot desulfurization of peptide *Ac*-Cys-Phe-Arg-Ala-Leu-$^{\alpha}$*COOH* (1) with ultrasound-induced desulfurization (USID) methodology 13
7. One-pot total chemical synthesis of Ubiquitin 14
7.1. Synthesis of ubiquitin peptide segment Met^{1}-Phe^{45}-$^{\alpha}$*COSR* (9)..14
7.2. Synthesis of ubiquitin peptide segment Cys^{46}-Gly^{76}-$^{\alpha}$*COOH* (10) 15
7.3. Native chemical ligation of ubiquitin peptide segments 9 and 10 followed by one-pot desulfurization 15
7.4. Characterization of Ubiquitin protein (12) by circular dichroism..16
7.5. Synthesis of Ubiquitin peptide segment Met^{1}-Phe^{45}- $^{\alpha}CONHNH_2$ *(13)* 16
7.6. Native chemical ligation of ubiquitin peptides 13 and 10 using Knorr pyrazole synthesis strategy to active hydrazide of segment 13 followed by one-pot desulfurization..17
7.7. Characterization of Ubiquitin protein 12 by circular dichroism 17
8. One-pot synthesis of collagen polypeptide Gly^{1}-Arg^{99}-$^{\alpha}$*COOH* (30) via five consecutive ligations followed by desulfurization 18
8.1. Synthesis of collagen peptide segment Gly^{1}-Lys^{20}- $^{\alpha}$*COSR* (16) 18
8.2. Synthesis of collagen peptide segment *Fmoc*-Cys^{21}-Gly^{40}- $^{\alpha}$*COSR* (18) 18
8.3. Synthesis of collagen peptide segment *Fmoc*-Cys^{41}-Ala^{56}- $^{\alpha}$*COSR* (20)..19
8.4. Synthesis of collagen peptide segment *Fmoc*-Cys^{57}-Gly^{73}- $^{\alpha}$*COSR* (22) 20
8.5. Synthesis of collagen peptide segment *Fmoc*-Cys^{74}-Gly^{85}- $^{\alpha}$*COSR* (24) 21
8.6. Synthesis of collagen peptide segment Cys^{86}-Arg^{99}- $^{\alpha}$*COOH* (25) 21
8.7. One-pot six-segment ligation of collagen peptide segments 16, 18, 20, 22, 24, and 25 followed by one-pot desulfurization..22
9. One-pot total chemical synthesis of Barstar A protein..23
9.1. Synthesis of Barstar A segment Lys^{1}-Leu^{24} - $^{\alpha}$*COSR* (32)..23
9.2. Synthesis of Barstar A segment *Fmoc*-Cys^{25}-Gly^{66}- $^{\alpha}$*COSR* (34) 24
9.3. Synthesis of Barstar A segment Cys^{67}-Ser^{89}- $^{\alpha}$*COOH* (35) 25
9.4. One-pot three-segment ligation of Barstar A peptide segments 32, 34, and 35 followed by one-pot desulfurization 25
9.5. Characterization of Barstar A protein (38) by circular dichroism..26
9.6. Inhibition of ribonuclease activity of Barnase protein with enhancement of Barstar A (38) concentration..26
References 27

1. General Methods

1.1. Reagents

N,N-Diisopropylethylamine (DIEA), Tris(2-carboxyethyl)phosphine hydrochloride (TCEP), Guanidine hydrochloride (Gdn.HCl), Ethyl cyanohydroxyiminoacetate (Oxyma), 4-mercaptophenylacetic acid (MPAA) and all the N^{α}-Fmoc protected amino acids were obtained from Chem-Impex International, USA. The sidechain protecting groups used were Asp(OtBu), Glu(OtBu), Asn(trt), Arg(Pbf), Ser(tBu), Thr(tBu), Tyr(tBu) and Fmoc-(Dmb)Gly-OH. Fmoc-Cys(trt)-OH was purchased from Gyros Protein Technologies. *N,N*-Dimethylformamide (DMF), dichloromethane(DCM), peptide synthesis grade acetonitrile (CH_3CN), diethyl ether, HPLC grade *N,N′*-diisopropylcarbodiimide (DIC), and trifluoroacetic acid (TFA) were purchased from SRL chemicals India. The HPLC grade acetonitrile (CH_3CN) for peptide purification was purchased from Thermofisher Scientific, India. Piperidine was obtained from AVRA chemicals, India. 2-Chlorotrityl chloride (2-Cl-(Trt)-Cl) resin was purchased from Supra Sciences, India. The Rink amide aminomethyl resin (polystyrene resin with 1% cross-linked with divinylbenzene) was obtained from Supra Science Private Limited, India. Bromoacetic acid was obtained from SRL Chemicals, India. Chloroacetic acid and bromoacetamide were purchased from TCI Chemicals. Sodium 2-mercaptoethanesulfonate (MESNa) and all other common reagents were purchased from Sigma-Aldrich and were of the purest grade available. Yeast RNA (Product No 10109223001) was purchased from Sigma-Aldrich.

1.2. Reverse-phase HPLC and LC-MS analysis

Analytical reverse-phase (RP) HPLC was performed on an Agilent HPLC instrument using an Agilent zorbax SB-C3 (5 µm), 4.6×150 mm reverse-phase silica column at a flow rate of 0.9 mL/min using a linear gradient of 10-54% solvent B in solvent A over 22 min or 10-64% solvent B in solvent A over 27 min or 20-80% solvent B in solvent A over 30 min at 40 °C (solvent A= 0.1% TFA in H_2O; solvent B = 0.08% TFA in acetonitrile). The UV absorbance of the column eluent was monitored at 214 nm wavelength. The peptide masses were measured across the peak by online LC-MS using an Agilent 1290 infinity II/6530 Q-TOF LC/MS instrument. The deconvolution of the charge states of the observed mass was carried out using Agilent MassHunter Qualitative Analysis software (version B.07.00), and the deconvoluted mass of the most abundant isotopologue has been reported with an uncertainty of ± 0.02 Da unless stated otherwise. Calculated masses were based on average isotope composition or based on the most abundant isotopologue mass determined from the isotopic distribution provided by Agilent MassHunter Qualitative Analysis software.

Preparative reverse phase HPLC (RP-HPLC) of crude peptides was performed with a Waters 1525 preparative HPLC system using Phenomenox C18 (5 µm, 100 Å, 10 x 250 mm) or Agilent ZORBAX-SB C3 (5 µm, 300 Å, 9.4 x 250 mm) columns at 40 °C using an appropriate shallow gradient of increasing concentration of solvent B (0.08% TFA in acetonitrile) in solvent A (0.1% TFA in water) at a flow rate of 5 mL/min. Fractions containing the purified target peptide were identified by ESI-MS. Selected pure fractions were then pooled and lyophilized.

1.3. A general protocol for machine-assisted Fmoc-SPPS

All peptides were synthesized using an automated peptide synthesizer (Tribute-UV/IR from Protein Technologies, USA). Fmoc-SPPS was carried out following reported[1] protocol with minor modifications, using amino acids (AA) (0.25 M), DIC (0.25 M) as a coupling reagent and Oxyma (0.25 M) with DIEA (0.025 M) as additives. Peptides were synthesized on Rink amide aminomethyl resin with a loading capacity of 0.5-0.6 mmol/g. Cysteine and Arginine were coupled for 15 min at room temperature followed by 5 min at 50 °C and all other amino-acid coupling was performed for 10 min at 65 °C under an N_2 atmosphere with vortex mixing. All other amino acid couplings on the 2-2-Cl-(Trt)-resin were performed for 6 min at 50 °C under N_2 atmosphere with vortex mixing. For the synthesis of peptide sequences containing Asp-Gly, which is prone to aspartimide formation during Fmoc-SPPS at elevated temperatures, Fmoc-(Dmb)Gly-OH was used instead of Fmoc-Gly-OH. For the synthesis of C-terminal hydrazide peptide, hydrazine was coupled by adding 10% (vol/vol) hydrazine in DMF and

gently agitating for 30 min.[2] The unreacted functional group on 2-Cl-(Trt)-resin was capped using 5% MeOH (vol/vol) in DMF. The coupling of amino acids on NH_2NH-2-Cl-(Trt)-resin was performed in a peptide synthesizer following the coupling protocol mentioned above. Fmoc deprotection after every coupling cycle was carried out by 20% piperidine treatment at 50 °C. After synthesis, the peptides were cleaved from the resin using TFA (85%), Phenol (5%), TIPS (2.5%), Water (5%) and DODT (2.5%) as a cleavage cocktail. After cleavage, the TFA was evaporated under N_2 flow inside a well-ventilated fume hood. The cleaved peptide was precipitated and washed with cold diethyl ether. Dry crude peptides were either dissolved in 6 M Gu.HCl and loaded directly onto a preparative HPLC column for purification or used for successive reactions without further purification.

1.4. A general protocol for manual Fmoc-SPPS

All peptides were synthesized manually in a glass reaction vessel. Fmoc-SPPS was carried out following the reported[1] protocol with minor modifications, using amino acids (AA) (0.4 M), HBTU (0.4 M) as a coupling reagent, and DIEA (0.8 M) as an additive. Cysteine and Arginine were coupled for 30 min at room temperature. All other amino acid couplings on either 2-Cl-(Trt)-resin or Fmoc-protected Rink-Amide aminomethyl resin were performed for 15 min at RT with manual stirring in between. For the synthesis of peptide sequences containing Asp-Gly, which is prone to aspartimide formation during Fmoc-SPPS, Fmoc-(Dmb)Gly-OH was used instead of Fmoc-Gly-OH. N-terminal amine was acylated using AcOH (0.5 M), and DIC (0.5 M) twice at RT for 20 minutes each whenever required. For the synthesis of C-terminal hydrazide peptide, hydrazine was coupled by adding 10% (vol/vol) hydrazine in DMF and gently agitating for 30 min.[2] The unreacted functional group on 2-Cl-(Trt)-resin was capped using 5% MeOH (vol/vol) in DMF. The coupling of amino acids on NH_2NH-2-Cl-(Trt)-resin was performed in a peptide synthesizer following the coupling protocol mentioned above. Fmoc deprotection after every coupling cycle was carried out twice by 20% piperidine treatment for 5 minutes each at RT. After synthesis, the peptides were cleaved from the resin using TFA (85%), Phenol (5%), TIPS (2.5%), Water (2.5%), Thioanisole (2.5%) and DODT (2.5%) as a cleavage cocktail. After cleavage, the TFA was evaporated under N_2 flow inside a well-ventilated fume hood. The cleaved peptide was precipitated and washed with cold diethyl ether. Dry crude peptides were either dissolved in 6 M Gu.HCl and loaded directly onto a preparative HPLC column for purification or used for successive reactions without further purification.

1.5. Circular dichroism

The CD spectrum of the chemically synthesized ubiquitin and Barstar A was recorded using a Jasco CD spectrometer. The proteins were folded as mentioned in sections (**6.4**, **6.6**, and **8.5**) and transferred to a Hellma cuvette of path length 1 mm. The spectrum was measured at room temperature (25 °C) over the wavelength range of 185-260 nm with 0.5 nm data interval and averaging of three scans. Molar ellipticity was calculated using the following equation,

Mean residual ellipticity (deg.cm^2.dmol^{-1}) = $\frac{CD\ value\ (mdeg)*Mw}{10*c*l*Nr}$

where Mw is the molecular weight of the protein, c is concentration in g/L, l is the path length of the cuvette in cm and Nr is the number of amino acid residues in the protein. The concentration of proteins was determined by IMPLEN Spectrophotometer (NanoPhotometer® NP80) at 280 nm using the extinction coefficients derived from the protein sequence.

2. Model peptide synthesis

2.1. Synthesis of *Ac*-Cys-Phe-Arg-Ala-Leu-$^{\alpha}$*COOH* (1)

The model peptide *Ac*-Cys-Phe-Arg-Ala-Leu-$^{\alpha}$*COOH* (**1**) was synthesized on 2-Cl-(Trt)-resin (substitution = 0.6 mmol/g) by stepwise Fmoc chemistry SPPS on a 0.3 mmol scale manually in a glass reaction vessel at RT (see **Section 1.4** for the peptide synthesis protocol). The amino group of the N-terminal cysteine residue was acetylated on the resin. Purification using preparative HPLC gave 66.5 mg (102.25 µmol, 34.1% yield) of the pure peptide **1** (**Figure S1**). Observed mass (ESI-MS): 650.33 Da (deconvoluted most abundant isotopologue); calculated mass: 650.32 Da (most abundant isotopologue).

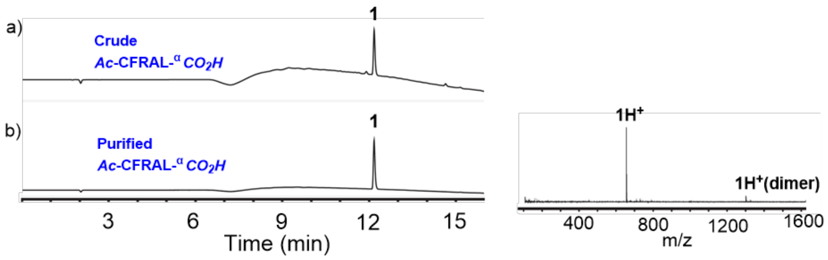

Figure S1. Analytical RP-HPLC profile (λ = 214 nm) together with ESI-MS data (right) of (a) crude peptide *Ac*-Cys-Phe-Arg-Ala-Leu-$^{\alpha}$*COOH* (**1**) (b) purified peptide *Ac*-Cys-Phe-Arg-Ala-Leu-$^{\alpha}$*COOH* (**1**) synthesized using 2-Cl-(Trt)-resin. Linear gradient 15-35% of B over 10 min including 4 min equilibration time using Agilent Zorbax SB-C3, 5 µm, 4.6 x 150 mm, 120Å LC column with 0.9 mL/min flow rate was used for the chromatographic separation. Purification was performed using a linear gradient of 10%-50% buffer B in buffer A over 40 min with a flow rate of 5 mL/min at 40 °C (buffer A = 0.1% TFA in water; buffer B = 0.08% TFA in acetonitrile) using a C18, 10 x 250 mm, preparative HPLC column (Phenomenex, 100 Å, 5 µm).

2.2. Synthesis of model peptide Ala-Pen-Gly-Phe-Arg-Ala-Leu-$^{\alpha}$*CONH$_2$* (1α)

The desired peptide Ala-Pen-Gly-Phe-Arg-Ala-Leu-$^{\alpha}$*CONH$_2$* (**1α**) was obtained by machine-assisted SPPS at elevated temperature (*see* **Section 1.3** for the peptide synthesis protocol). Stepwise synthesis of the peptide was carried out on Fmoc deprotected Rink-Amide aminomethyl resin (substitution = 0.5 mmol/g) in a 0.2 mmol scale. Global deprotection and cleavage of the peptide from the resin using a TFA cocktail followed by HPLC purification gave 70 mg (91.69 µmol, 45.87% yield) of the desired peptide Ala-Pen-Gly-Phe-Arg-Ala-Leu-$^{\alpha}$*CONH$_2$*. Observed mass **(ESI-MS)**: 763.42 Da (deconvoluted most abundant isotopologue, monoisotopic), calculated mass: 763.41 Da (monoisotopic). (**Figure S2**).

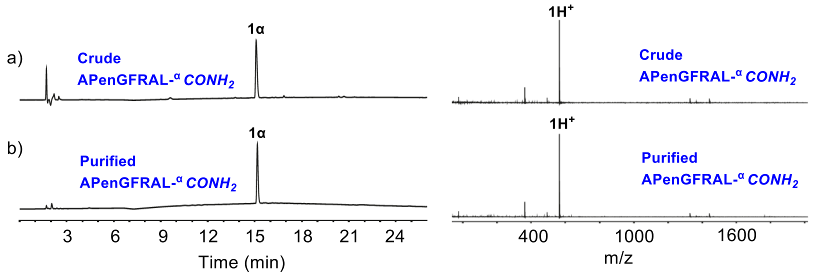

Figure S2. Analytical RP-HPLC profile (λ = 214 nm) together with ESI-MS data (right) for the synthesis of peptide Ala-Pen-Gly-Phe-Arg-Ala-Leu-$^{\alpha}$*CONH$_2$* (**1α**). (a) Chromatogram for the crude peptide Ala-Pen-Gly-Phe-Arg-Ala-Leu-$^{\alpha}$*CONH$_2$* (**1α**). (b) Chromatogram for the purified peptide Ala-Pen-Gly-Phe-Arg-Ala-Leu-$^{\alpha}$*CONH$_2$* (**1α**). Linear gradient 01%-61% of B over 20 min including 4 min equilibration using Agilent Zorbax SB-C3, 5 µm, 4.6 x 150 mm, 120Å LC column with 0.9 mL/min flow rate was used for all the chromatographic separation. Purification was performed using a linear gradient of 5% - 45% buffer B in buffer A over 40 min with a flow rate of 5 mL/min at 40 °C (buffer A = 0.1% TFA in water; buffer B = 0.08% TFA in acetonitrile) using a C18, 10 x 250 mm, preparative HPLC column (Phenomenex proteo, 100 Å, 5 µm).

2.3. Synthesis of model peptide Ser-Thr-Phe-Thr-Lys-Ser-Pro-Cys-$^{\alpha}$*CONH$_2$* (2)

The desired peptide Ser-Thr-Phe-Thr-Lys-Ser-Pro-Cys-$^{\alpha}$*CONH$_2$* (**2**) was obtained by machine-assisted SPPS at elevated temperature (*see* **Section 1.3** for the peptide synthesis protocol). Stepwise synthesis of the peptide was carried out on Fmoc deprotected Rink-Amide aminomethyl resin (substitution = 0.5 mmol/g) in a 0.2 mmol scale. Global deprotection and cleavage of the peptide from the resin using a TFA cocktail followed by HPLC purification gave 56 mg (64.48 µmol, 32.25% yield) of the desired peptide Ser-Thr-Phe-Thr-Lys-Ser-Pro-Cys-$^{\alpha}$*CONH$_2$* **(2)**. Observed mass **(ESI-MS)**: 868.42 Da (deconvoluted most abundant isotopologue), calculated mass: 868.41 Da (most abundant isotopologue). (**Figure S3**).

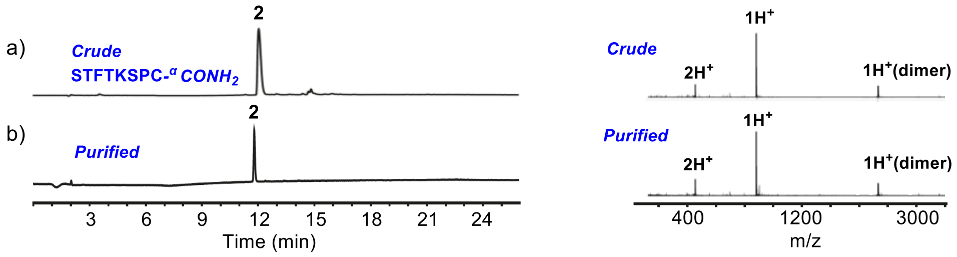

Figure S3. Analytical RP-HPLC profile (λ = 214 nm) together with ESI-MS data (right) for the synthesis of peptide Ser-Thr-Phe-Thr-Lys-Ser-Pro-Cys-$^{\alpha}$*CONH$_2$* (**2**). (a) Chromatogram for the crude peptide Ser-Thr-Phe-Thr-Lys-Ser-Pro-Cys-$^{\alpha}$*CONH$_2$* (**2**). (b) Chromatogram for the purified peptide Ser-Thr-Phe-Thr-Lys-Ser-Pro-Cys-$^{\alpha}$*CONH$_2$* (**2**). Linear gradient 01%-61% of B over 20 min including 4 min equilibration using Agilent Zorbax SB-C3, 5 µm, 4.6 x 150 mm, 120Å LC column with 0.9 mL/min flow rate was used for all the chromatographic separation. Purification was performed using a linear gradient of 5% - 45% buffer B in buffer A over 40 min with a flow rate of 5 mL/min at 40 °C (buffer A = 0.1% TFA in water; buffer B = 0.08% TFA in acetonitrile) using a C18, 10 x 250 mm, preparative HPLC column (Phenomenex proteo, 100 Å, 5 µm).

2.4. Synthesis of model peptide Leu-Tyr-Arg-Ala-Leu-$^{\alpha}$*COSR* (4)

The corresponding hydrazide peptide Leu-Tyr-Arg-Ala-Leu-$^{\alpha}$*CONHNH$_2$* (**3**) was first synthesized using NH_2NH-2-Cl-(Trt)-resin (substitution = 0.6 mmol/g) by stepwise Fmoc chemistry SPPS (0.2 mmol scales) in an automated peptide synthesizer (see **Section-1.3** for the peptide synthesis protocol). After global deprotection using a TFA cocktail, the peptide hydrazide was precipitated using cold diethyl ether. The mass of the crude peptide **3** was confirmed by LC-MS (**Figure S4a**); Observed mass (ESI-MS): 648.41 Da (deconvoluted most abundant isotopologue, monoisotopic); calculated mass: 648.40 Da (monoisotopic). Crude peptide **3** (~110 mg, ~0.2 mmol) was then dissolved in 10 mL of aqueous phosphate buffer (0.2 M) containing 6 M Gu.HCl at pH 3.0 and incubated at -16.5 °C (using Julabo). After 15 min, 1.0 mL of aqueous $NaNO_2$ (0.5 M) was added to the solution of peptide **3** and gently agitated for 15 min at -16.5 °C. Afterward, 10 mL of 0.2 M MESNa containing 0.2 M aqueous phosphate buffer and 6 M Gu.HCl at pH 6.3 was mixed into the oxidized solution of peptide **3** and the temperature was raised to room temperature. The MESNa exchange was complete within 30 min as monitored by LCMS (**Figure S4b**). Finally, the pH of the resulting reaction mixture was adjusted to 3.5 before purification. Purification using preparative HPLC afforded 51 mg (67.25 µmol, 33.62% yield) of the desired MESNa exchanged purified peptide **4** (**Figure S4c**). Observed mass (ESI-MS): 758.35 Da (deconvoluted most abundant isotopologue); calculated mass: 758.34 Da (most abundant isotopologue).

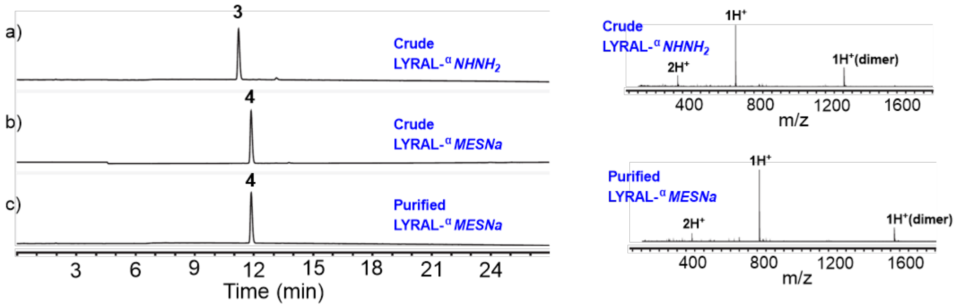

Figure S4. Analytical RP-HPLC profile (λ = 214 nm) together with ESI-MS data (right) for the synthesis of peptide Leu-Tyr-Arg-Ala-Leu-$^{\alpha}$*COSR* (**4**). (a) Chromatogram for the crude peptide Leu-Tyr-Arg-Ala-Leu-$^{\alpha}$*CONHNH$_2$* (**3**). (b) Chromatogram for the MESNa exchanged product Leu-Tyr-Arg-Ala-Leu-$^{\alpha}$*COSR* (**4**) from the crude peptide **3**. (c) Chromatogram for the purified peptide Leu-Tyr-Arg-Ala-Leu-$^{\alpha}$*COSR* (**4**). R = –$^{\alpha}$*COCH$_2$CH$_2$SO$_3$Na* Linear gradient 10%-54% of B over 22 min including 4 min equilibration using Agilent Zorbax SB-C3, 5 µm, 4.6 x 150 mm, 120Å LC column with 0.9 mL/min flow rate was used for all the chromatographic separation. Purification was performed using a linear gradient of 5%-45% buffer B in buffer A over 40 min with a flow rate of 5 mL/min at 40 °C (buffer A = 0.1% TFA in water; buffer B = 0.08% TFA in acetonitrile) using a C18, 10 x 250 mm, preparative HPLC column (Phenomenex proteo, 100 Å, 5 µm).

2.5. Synthesis of model peptide Cys-Leu-Tyr-Leu-Ala-Ala-$^{\alpha}$*COOH* (5)

The peptide Cys-Leu-Tyr-Leu-Ala-Ala-$^{\alpha}$*COOH* (**5**) was obtained by machine-assisted SPPS at elevated temperature (see **Section 1.3** for the peptide synthesis protocol). Stepwise peptide synthesis was carried out on 2-CTC-resin (substitution = 0.5 mmol/g) with a 0.2 mmol scale. Global deprotection and cleavage of the peptide from the resin using a TFA cocktail followed by HPLC purification gave 55.72 mg (85.41

µmol, 42.7% yield) of the desired peptide **5**. Observed mass (ESI-MS): 652.33 Da (deconvoluted most abundant isotopologue); calculated mass: 652.32 Da (most abundant isotopologue) (**Figure S5**).

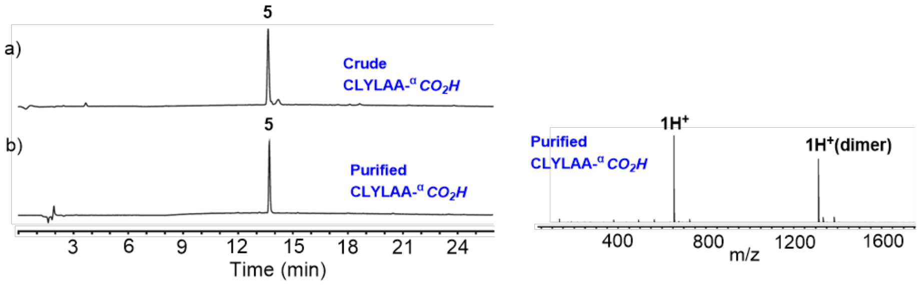

Figure S5. Analytical RP-HPLC profile (λ = 214 nm) together with ESI-MS data (right) for the synthesis of peptide Cys-Leu-Tyr-Leu-Ala-Ala-$^{\alpha}$COOH (**5**). (a) Chromatogram for the crude peptide Cys-Leu-Tyr-Leu-Ala-Ala-$^{\alpha}$*COOH* (**5**). (b) Cys-Leu-Tyr-Leu-Ala-Ala-$^{\alpha}$*COOH* (**5**). Linear gradient 10%-54% of B over 22 min including 4 min equilibration using Agilent Zorbax SB-C3, 5 µm, 4.6 x 150 mm, 120Å LC column with 0.9 mL/min flow rate was used for all the chromatographic separation. Purification was performed using a linear gradient 10%-50% buffer B in buffer A over 40 min with a flow rate of 5 mL/min at 40 °C (buffer A = 0.1% TFA in water; buffer B = 0.08% TFA in acetonitrile) using a C18, 10 x 250 mm, preparative HPLC column (Phenomenex proteo, 100 Å, 5 µm).

3. Optimization of the reactions of MPAA with alkylating reagents

3.1. Rate of reaction between MPAA and alkylating reagents at pH 5 and 6.6

To check the feasibility of the reaction between MPAA (**) and three different alkylating reagents, bromoacetic acid, bromoacetamide, and chloroacetic acid, two different pH 5 and 6.6 were tested. In the first experiment, MPAA (3.4 mg, 20 mM) and TCEP (5.7 mg, 20 mM) were dissolved together in 1 mL of 6 M Gu.HCl, 0.2 M phosphate buffer, and the pH was adjusted to 5.0. Out of 1 mL, 200 µL solution was eluted into three different vials for three different reactions. Separately, 250 mM stock solutions of bromoacetic acid, bromoacetamide, and chloroacetic acid were prepared in the same buffer as above with the final pH of 5.0 and then 22 µL from the stock solution was added to the three vials to make the final concentration of the alkylating reagents approximately 25 mM, respectively. The identity of the MPAA alkylated products (*, #) at different time points, 1 min, 2.5 min, 5 min, 30 min, 60 min, 180 min, 360 min, and 960 min for all three reactions at pH 5.0 was confirmed by HPLC and ESI-MS (main text **Figure 1-C, Figure S6-A**). As anticipated, bromoacetic acid and bromoacetamide alkylated MPAA within 15 minutes at pH 5.0. Similarly, the above experiment was repeated at pH 6.6. The identity of the MPAA alkylated products (*, #) at different time points, 1 min, 2.5 min, 5 min, 30 min, 60 min, and 120 min for all three reactions was monitored by RP-HPLC (**Figure S6-B**). The percentage of conversion was calculated from the area under the curve obtained from the 214 nm UV absorption spectra in RP-HPLC. The plot showed that bromoacetic acid and bromoacetamide alkylated the total MPAA within 2 minutes at pH 6.6 whereas chloroacetic acid took 2 hours (main text **Figure 1-D, Figure S6-B**).

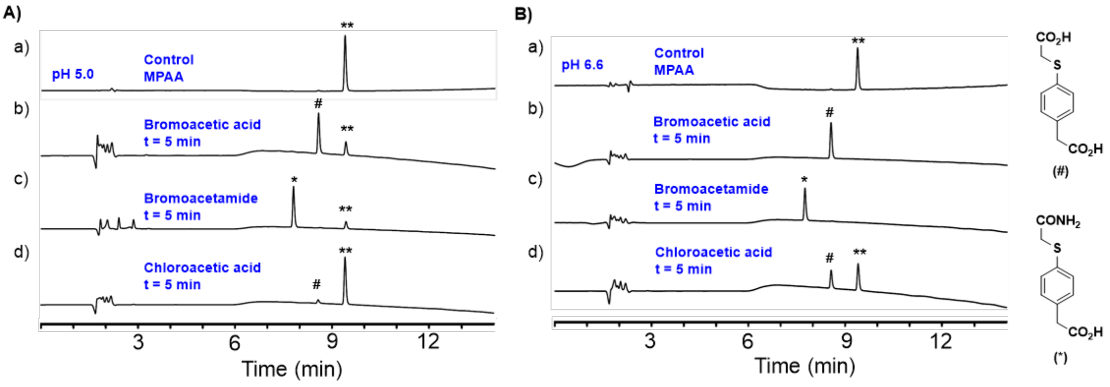

Figure S6. Analytical RP-HPLC profile (λ = 214 nm) for the reaction of MPAA (**) with alkylating reagents at different pH A) Chromatogram for the reaction at pH 5.0 (a) only MPAA (b) in the presence of 20 mM bromoacetic acid after 5 minutes (c) in the presence of 20 mM bromoacetamide after

5 minutes (d) in the presence of 20 mM chloroacetic acid after 5 minutes B) Chromatogram for the reaction at pH 6.6 (a) only MPAA (b) in the presence of 20 mM bromoacetic acid after 5 minutes (c) in the presence of 20 mM bromoacetamide after 5 minutes (d) in the presence of 20 mM chloroacetic acid after 5 minutes. Linear gradient 10%-100% of B over 10 min including 4 min equilibration using Agilent Zorbax SB-C3 5 µm 4.6 x 150 mm, 120Å LC column with 0.9 mL/min flow rate was used for all the chromatographic separation. The '**' indicates MPAA and '#' and '*' indicates the alkylated MPAA as shown in the structure (right).

3.2. Evaluating the specificity of bromoacetamide towards MPAA in the presence of model peptide 1

To check the selectivity of aryl thiol MPAA (**) towards bromoacetamide in the presence of cysteine-containing model peptide **1**, we performed an experiment based on section 3.1. The schematic of the reaction is shown in **Figure S7-A**. Peptide **1** (0.65 mg, 1mM) along with MPAA (3.4 mg, 20 mM) and TCEP (5.7 mg, 20 mM) were dissolved together in 1 mL of 6 M Gu.HCl, 0.2 M phosphate buffer at a final pH of 6.8. Out of 1 mL, 200 µL solution was eluted into three different vials for three different reactions. Separately, the concentrated stock solutions of bromoacetamide (2 M, 3 M, and 4 M) were prepared in the same buffer (6 M Gu.HCl, 0.2 M phosphate) and the pH was adjusted to 6.6. From the three stock solutions, 2 µL, 3 µL, and 4 µL were added to the above three vials at the same time to make the final concentrations of the bromoacetamide approximately 20 mM, 30 mM, and 40 mM, respectively. The final pH of the three reactions was 6.58, 6.60, and 6.57. The progress of the three reactions at different time intervals was monitored by RP-HPLC and the percentage of conversion was calculated from the area under the curve obtained from the 214 nm UV absorption spectra in RP-HPLC (**Figure S7-B**). Observed mass of **1** (ESI-MS): 650.33 Da (deconvoluted most abundant isotopologue); calculated mass of **1**: 650.32 Da (most abundant isotopologue). Observed mass of **1'** (ESI-MS): 707.36 Da (deconvoluted most abundant isotopologue); calculated mass of **1'**: 707.34 Da (most abundant isotopologue). As shown in **Figure S7-C,** the higher equivalence of bromoacetamide as compared to MPAA, increases the percentage of by-product **1'**. To minimize the formation of by-product **1'** to a minimum, bromoacetamide equivalence should be either exact or slightly less (0.95 eq.) of MPAA.

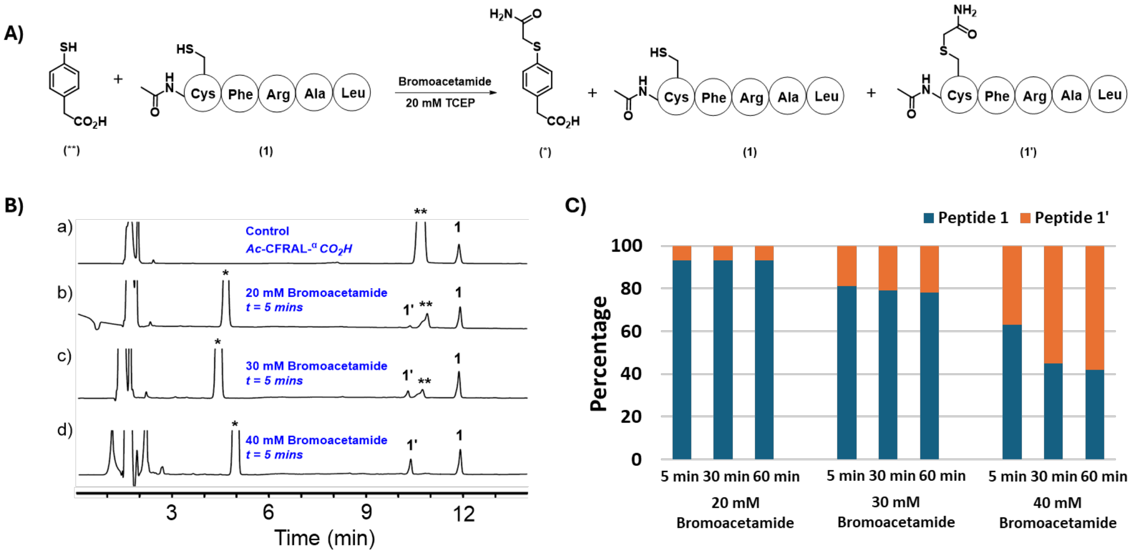

Figure S7. A) Schematic of the reaction of 20 mM MPAA (**) with different concentrations of bromoacetamide in the presence of 1 mM peptide **1** in the buffer (6 M Gu.HCl, 0.2 M PB, 20 mM TCEP) B) Analytical RP-HPLC profile (λ = 214 nm) monitoring the reaction (a) only MPAA (b) in the presence of 20 mM bromoacetamide after 5 minutes (c) in the presence of 30 mM bromoacetamide after 5 minutes (d) in the presence of 40 mM bromoacetamide after 5 minutes C) stacked column plot showing the ratio of the formation of peptide **1** (blue color) to by-product **1'** (orange color) at different times points 5 min, 30 min, and 60 min for 20mM, 30mM, and 40mM bromoacetamide concentration each. The percentage of conversion was calculated from the area under the curve obtained from the 214 nm UV absorption spectra in RP-HPLC. Linear gradient 15%-35% of B over 10 min including 3 min equilibration using Agilent Zorbax SB-C3 5 µm 4.6 x 150 mm LC, 120Å column with 0.9 mL/min flow rate was used for all the chromatographic separation. The '**' indicates MPAA and (*) indicates alkylated MPAA.

3.3. Effect of alkyl thiol additives on the alkylation (by-product) of peptide 1

To reduce the formation of by-product (**1'**) to a minimum, we studied the effect of the presence of different alkyl thiols during the quenching of MPAA with bromoacetamide. Alkyl thiols such as MESNa, cysteine, acetylcysteine, cysteamine, and 3-mercapto-1-propanesulfonic acid (MPSNa) were studied which can act as H-donors for the desulfurization process. Peptide **1** (0.78 mg, 1 mM) along with MPAA (4.0 mg,

20 mM) and TCEP (6.8 mg, 20 mM) were dissolved together in 1.2 mL of 6 M Gu.HCl, 0.2 M phosphate buffer at a final pH of 6.7. Out of 1 mL, 200 µL solution was eluted into six different Eppendorf vials. With one vial working as a control with no additive alkyl thiol, to remaining five vials, MESNa (3.28 mg, 100 mM), cysteine (2.42 mg, 100 mM), N-acetyl cysteine (3.26 mg, 100 mM), cysteamine (2.27 mg, 100 mM), and 3-mercapto-1-propanesulfonic acid (3.6 mg, 100 mM) were added, respectively and the pH was adjusted to 6.6. To each of these five vials, 20 mM bromoacetamide (0.57 mg, 4.0 µmoles, 20 mM) dissolved in 10 µL of water was added at the same time. The progress of all five reactions after 5 minutes was monitored by RP-HPLC and the percentage of conversion was calculated from the area under the curve obtained from the 214 nm UV absorption spectra in RP-HPLC. As shown in the plot (**Figure S8-B**), N-acetylcysteine reduced the formation of the alkylated peptide by-product **1'** to 2.5% from 8%. The reaction was monitored by RP-HPLC and ESI-MS. Observed mass of **1** (ESI-MS): 650.33 Da (deconvoluted monoisotopic peak); calculated mass of **1**: 650.32 Da (monoisotopic). Observed mass of **1'** (ESI-MS): 707.36 Da (deconvoluted most abundant isotopologue); calculated mass of **1'**: 707.34 Da (most abundant isotopologue).

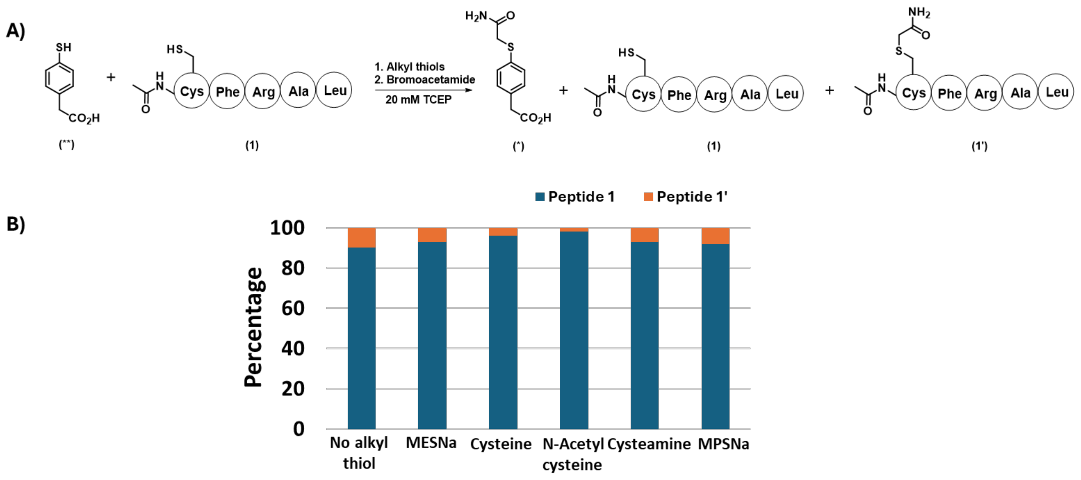

Figure S8. A) Schematic of the reaction of 20 mM MPAA (**) with 20 mM bromoacetamide in the presence of 100 mM alkyl thiols and 1 mM peptide **1** in the buffer (6 M Gu.HCl, 0.2 M PB, 20 mM TCEP) B) stacked column plot showing the ratio of the formation of peptide **1** (blue color) to by-product **1'** (orange color) at a time point of 5 min for 20mM bromoacetamide concentration in the presence of 100 mM of alkyl thiols, MESNa, cysteine, acetylcysteine, cysteamine, and 3-mercapto-1-propanesulfonic acid respectively. The percentage of conversion was calculated from the area under the curve obtained from the 214 nm UV absorption spectra in RP-HPLC. Linear gradient 15%-35% of B over 10 min including 3 min equilibration using Agilent Zorbax SB-C3, 5 µm, 4.6 x 150 mm, 120Å LC column with 0.9 mL/min flow rate was used for all the chromatographic separation.

3.4. Reaction of MPAA with bromoacetamide in the presence of N-acetyl cysteine and model peptide 2

To confirm the selectivity of MPAA for bromoacetamide over other reactive residues of the peptide at pH 6.7 for one-pot desulfurization process, we used model peptide Ser-Thr-Phe-Thr-Lys-Ser-Phe-Cys-$^{\alpha}$*CONH*$_2$ **(2)** which contains Ser, Lys as well as Cys residues containing nucleophilic side chains. To a solution of peptide **2** (1 mg, 1.15 µmol, 1 mM), MPAA (3.87 mg, 23.01 µmol, 20 mM), and TCEP (6.59 mg, 23.02 µmol, 20 mM) dissolved in 1.15 mL of ligation buffer (200 mM PB, 6 M Gu.HCl) was added solid N-acetylcysteine (18.76 mg, 115 µmol, 100 mM) and the pH was adjusted to 6.7. Separately, bromoacetamide (3.174 mg, 23.01 µmol, 20 mM) was dissolved in 20 µL of water and then added slowly (5 µL x 4) to the reaction mixture over 2 minutes. The reaction mixture was further stirred for an extra 2-3 minutes. The identity of all the peaks was confirmed by RP-HPLC analysis and the percentage of conversion was calculated from the area under the curve obtained from the 214 nm UV absorption spectra in RP-HPLC (**Figure S9**) (Observed mass of **2** (ESI-MS): 868.42 Da (deconvoluted most abundant isotopologue); calculated mass of **2**: 868.41 Da (most abundant isotopologue).

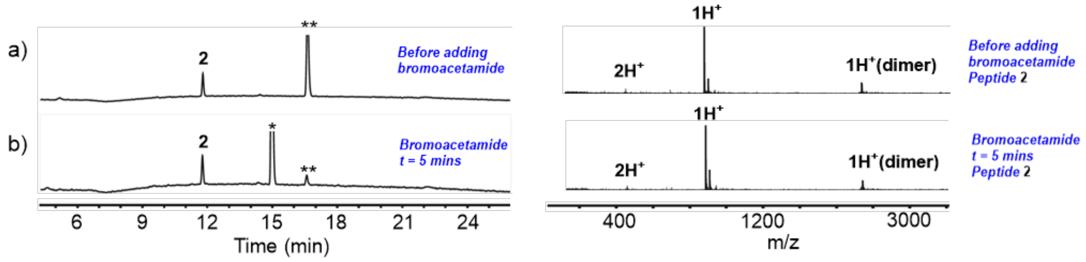

Figure S9. Analytical RP-HPLC profile (λ = 214 nm) and ESI-MS data (right) for the reaction of MPAA (**) with bromoacetamide in the presence of N-acetyl cysteine and peptide **2**. Chromatogram of the reaction containing 1 mM peptide **2**, 20 mM MPAA (**), and 100 mM N-acetylcysteine (a) before adding 20 mM bromoacetamide t = 0 min (b) after 5 minutes of adding 20 mM bromoacetamide. Linear gradient 01%-61% of B over 20 min including 4 min equilibration using Agilent Zorbax SB-C3 5 µm 4.6 x 150 mm, 120Å LC column with 0.9 mL/min flow rate was used for all the chromatographic separation. The '**' indicates MPAA and (*) indicates alkylated MPAA.

4. Ligation between two model peptides 4 and 5 followed by desulfurization under different conditions

4.1. Ligation between two model peptides 4 and 5 followed by direct desulfurization

To confirm whether the one-pot ligation-desulfurization can be performed directly in the presence of MPAA aryl thiol, we ligated a model peptide Leu-Tyr-Arg-Ala-Leu-$^{\alpha}$*COSR* (**4**) with Cys-Leu-Tyr-Leu-Ala-Ala-$^{\alpha}$*COOH* (**5**) followed by addition of desulfurizing reagents without any purification in-between. To a solution containing MPAA (46.4 mg, 275.8 µmol, 20 mM) and TCEP (79.1 mg, 275.8 µmol, 20 mM) in 13.2 mL ligation buffer (200 mM PB, 6 M Gdn.HCl, pH 7.0) solid peptide **4** (10 mg, 13.17 µmol, 1 mM) and peptide **5** (8.6 mg, 13.17 µmol, 1 mM) were added and the pH of the reaction mixture was adjusted to 6.85. The progress of the ligation reaction was monitored by reverse-phase HPLC. The ligation was completed within 5 h resulting in a clean conversion to the ligated product **6** (**Figure S10b**). After the ligation, MESNa (75 mM), TCEP (150 mM), and VA-044 (100 mM) were added to the above ligation mixture, and the pH of the reaction mixture was adjusted to 7.0. The reaction mixture was kept at 37°C. The progress of the reaction was monitored by reverse-phase HPLC and ESI-MS at different time intervals. As anticipated, even after 12 hours only 5% of the peptide **6** got desulfurized pointing out the need of selectively quenching MPAA for one-pot ligation-desulfurization (**Figure S10d**). Observed mass (**ESI-MS**):1236.73 Da (deconvoluted most abundant isotopologue); calculated mass: 1236.72 Da (most abundant isotopologue).

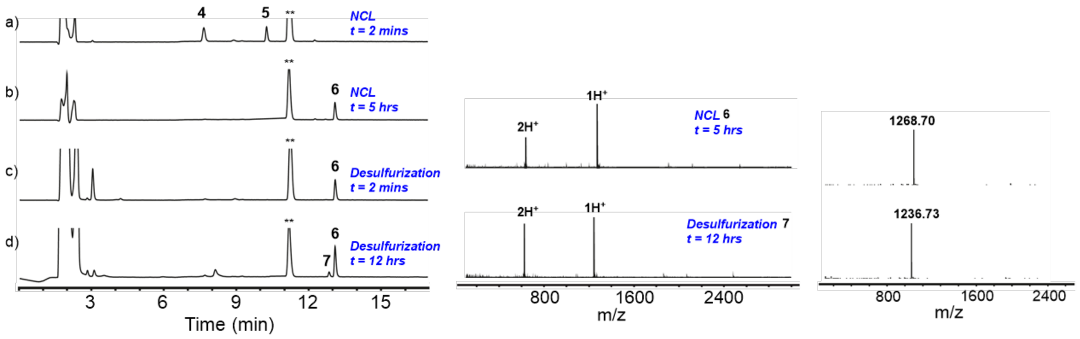

Figure S10. Analytical RP-HPLC profile (λ = 214 nm) together with ESI-MS data (right) of the ligation reaction followed by direct desulfurization. a) Chromatogram at 2 min after the addition of peptide **5** to the ligation buffer containing peptide **4** (b) Ligation was essentially completed within 5 h to give the ligated product **6**. (c) Chromatogram at 2 min after the initiation of desulfurization of peptide **6** (e) Chromatogram after 12 h of the desulfurization reaction where **7** is the desulfurized product. Linear gradient 10%-52% of B over 14 min including 4 min equilibration using Agilent Zorbax SB-C3 5 µm 4.6 x 150 mm LC column with 0.9 mL/min flow rate was used for all the chromatographic separation. The '**' indicates MPAA.

4.2. Ligation between two model peptides 4 and 5 followed by quenching of MPAA and desulfurization

To test the feasibility of the one-pot ligation-desulfurization strategy by quenching MPAA thiol via bromoacetamide, we ligated same model peptide Leu-Tyr-Arg-Ala-Leu-$^{\alpha}$*COSR* (**4**) with Cys-Leu-Tyr-Leu-Ala-Ala-$^{\alpha}$*COOH* (**5**) and quenched MPAA thiol with bromoacetamide in the presence of N-acetylcysteine followed by desulfurization. To a solution of MPAA (46.4 mg, 275.8 µmol, 20 mM) and TCEP (79.1 mg, 275.8 µmol, 20 mM) in ligation buffer (200 mM PB, 6 M Gu.HCl, pH 7.0), was added solid peptide **4** (10 mg, 13.17 µmol, 1 mM) and peptide **5** (8.6 mg, 13.17 µmol, 1 mM) and the pH of the reaction mixture was adjusted to 6.85. The progress of the ligation reaction was monitored by reverse-phase HPLC, and the ligation was essentially completed within 5 h resulting in a clean conversion to the ligated product **6** (main text, **Figure 2-B(b)**). After the ligation, N-acetyl cysteine (225 mg, 1.38 mmol, 100 mM) was added to the reaction mixture and the pH was adjusted to 6.6 followed by the dropwise addition of bromoacetamide (38.1 mg, 276 µmol, 20 mM) dissolved in 20 µL water over 2 minutes. The reaction was stirred at RT for an extra 2-3 minutes (trace amount of MPAA left unreacted). To this solution, TCEP (645 mg, 2.25 mmol, 150 mM) and VA-044 (523.3 mg 1.62 mmol, 100 mM) were added and the pH of the solution was adjusted to 7.0 and the

reaction mixture was kept at 37°C until the completion of the reaction. After the completion of the reaction, the pH was again reduced to 3.0. Purification of the reaction mixture by preparative HPLC gave 12.05 mg (09.75 µmol, 74.0 % yield) of the desired desulfurized ligated peptide **7.** The identity of the desired desulfurized ligated product **7** was confirmed by RP-HPLC and ESI-MS analysis (main text **Figure 2-B(f), C,** and **Figure S11**) (Observed mass (ESI-MS): 1236.73 Da (deconvoluted most abundant isotopologue); calculated mass: 1236.72 Da (most abundant isotopologue).

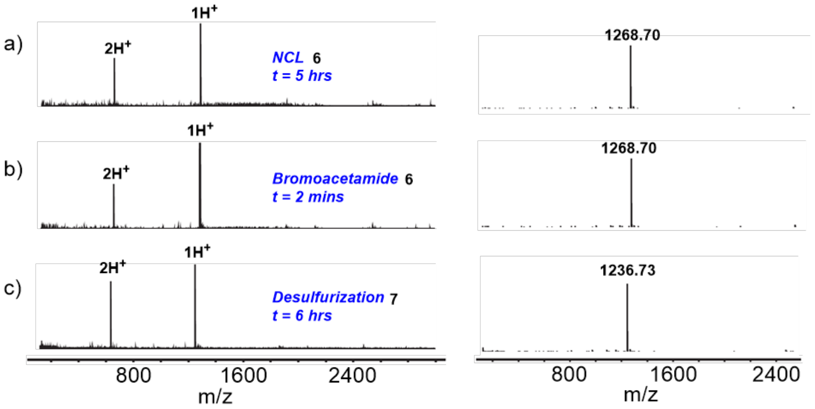

Figure S11. ESI-MS data of the ligation reaction followed by quenching of MPAA and one-pot desulfurization. ESI-MS data of the a) ligated polypeptide **6** after the ligation was over in 5 h (b) polypeptide **6** after 5 min of the addition of bromoacetamide (c) desulfurized polypeptide **7**. RP-HPLC chromatograms are shown in main text Figure 2-B. Linear gradient 10%-52% of B over 14 min including 4 min equilibration using Agilent Zorbax SB-C3 5 µm 4.6 x 150 mm LC column with 0.9 mL/min flow rate was used for all the chromatographic separation.

4.3. Ligation between two model peptides 4 and 5 followed by purification and desulfurization

To compare the yields between the standard ligation-purification-desulfurization process and one-pot ligation-desulfurization process, we ligated a model peptide Leu-Tyr-Arg-Ala-Leu-$^{\alpha}$*COSR* (**4**) with Cys-Leu-Tyr-Leu-Ala-Ala-$^{\alpha}$*COOH* (**5**) followed by reverse-phase HPLC purification and then desulfurization. To a solution of MPAA (46.4 mg, 275.8 µmol, 20 mM) and TCEP (79.1 mg, 275.8 µmol, 20 mM) in ligation buffer (200 mM PB, 6 M Gu.HCl, pH 7.0) was added solid peptide **4** (10 mg, 13.17 µmol, 1 mM) and peptide **5** (8.6 mg, 13.17 µmol, 1 mM) and the pH of the reaction mixture was adjusted to 6.85. The progress of the ligation reaction was monitored by reverse-phase HPLC, and the ligation was essentially completed within 5 h resulting in a clean conversion to the ligated product **6** (**Figure S12b**). After the ligation, the pH of the reaction was reduced to 3.0 and the peptide **6** was purified by reverse-phase preparative HPLC. Pure fractions were identified by HPLC chromatograms and were pooled and lyophilized. Purification of the reaction mixture by preparative HPLC gave 12.54 mg (09.88 µmol, 75.05 % yield) of the desired desulfurized ligated peptide **6** (Leu-Tyr-Arg-Ala-Leu-Cys-Leu-Tyr-Leu-Ala-Ala-$^{\alpha}$*COOH* (**Figure S12c**). Observed mass (**ESI-MS**): 1268.70 Da (deconvoluted most abundant isotopologue); calculated mass: 1268.69 Da (most abundant isotopologue). Pure peptide **6** (12.54 mg, 09.89 µmol) was dissolved in a solution of MESNa (75 mM), TCEP (150 mM), and VA-044 (100 mM) in the same ligation buffer as above. The pH of the reaction mixture was adjusted to 7.0 and the reaction mixture was kept at 37°C until the completion of the reaction. After the completion of the reaction, the pH was again reduced to 3.0. Purification of the reaction mixture by preparative HPLC gave 8.80 mg (07.12 µmol, 71.99 % yield) of the desired desulfurized ligated peptide **7** (Leu-Tyr-Arg-Ala-Leu-Cys-Leu-Tyr-Leu-Ala-Ala-$^{\alpha}$*COOH* (**Figure S12f**). Overall yield: 54.05% over two steps. Observed mass (**ESI-MS**): 1236.73 Da (deconvoluted most abundant isotopologue); calculated mass: 1236.72 Da (most abundant isotopologue).

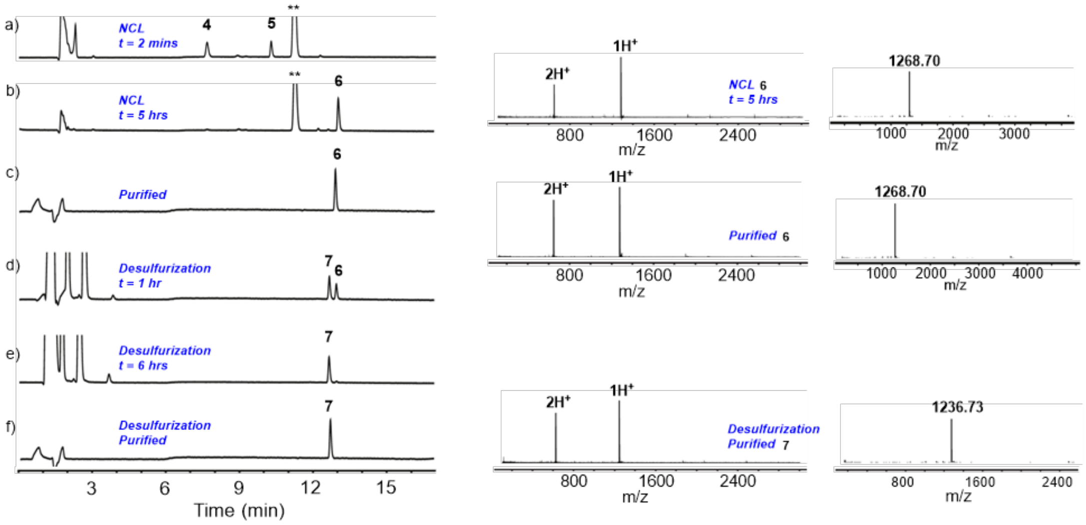

Figure S12. Analytical RP-HPLC profile (λ = 214 nm) together with ESI-MS data (right) of the ligation reaction followed by purification and desulfurization. (a) Chromatogram at 2 min after the addition of peptide **5** to the ligation buffer containing peptide **4** (b) Ligation was essentially completed within 5 h to give the ligated product **6**. (c) purified polypeptide **6** (d) chromatogram at 1 h after the initiation of desulfurization of peptide **6** (e) desulfurized polypeptide 7 (f) purified desulfurized polypeptide **7**. Linear gradient 10%-52% of B over 14 min including 4 min equilibration using Agilent Zorbax SB-C3 5 µm 4.6 x 150 mm LC column with 0.9 mL/min flow rate was used for all the chromatographic separation. The '**' indicates MPAA and (*) indicates alkylated MPAA.

5. Desulfurization of Ala-Pen-Gly-Phe-Arg-Ala-Leu-$^{\alpha}$*CONH$_2$* (1α) in the presence of MPAA

To confirm the selectivity of MPAA for bromoacetamide over penicillamine thiol group, we used model peptide Ala-Pen-Gly-Phe-Arg-Ala-Leu-$^{\alpha}$*CONH$_2$* (**1α**) containing penicillamine in the sequence. To a solution of peptide (7.6 mg, 1 mM), MPAA (33.6 mg, 20 mM), and TCEP (57.0 mg, 20 mM) dissolved in 1.0 mL of ligation buffer (200 mM PB, 6 M Gu.HCl) was added solid N-acetyl cysteine (163.9 mg, 100 mM) and the pH was adjusted to 6.7. Separately, bromoacetamide (30.4 mg, 22 mM) was dissolved in 200 µL of water and then added slowly (50 µL x 4) to the reaction mixture over 2 minutes. The reaction mixture was further stirred for an extra 2-3 minutes. To this solution (1.1 mL), solid VA-044 (355.3 mg, 100 mM) and TCEP (472.9, 150 mM) were added, and the pH was readjusted to 7.0. The reaction was incubated at 37°C for desulfurization until completion. After 8 hours, the desulfurized peptide was purified using reverse-phase chromatography yielding 6.1 mg (83.3%) pure peptide **1β.** The identity of all the peaks was confirmed by RP-HPLC analysis and the percentage of conversion was calculated from the area under the curve obtained from the 214 nm UV absorption spectra in RP-HPLC (**Figure S13**) (Observed mass of **1β** (ESI-MS): 731.42 Da (deconvoluted most abundant isotopologue); calculated mass of **2**: 731.44 Da (most abundant isotopologue).

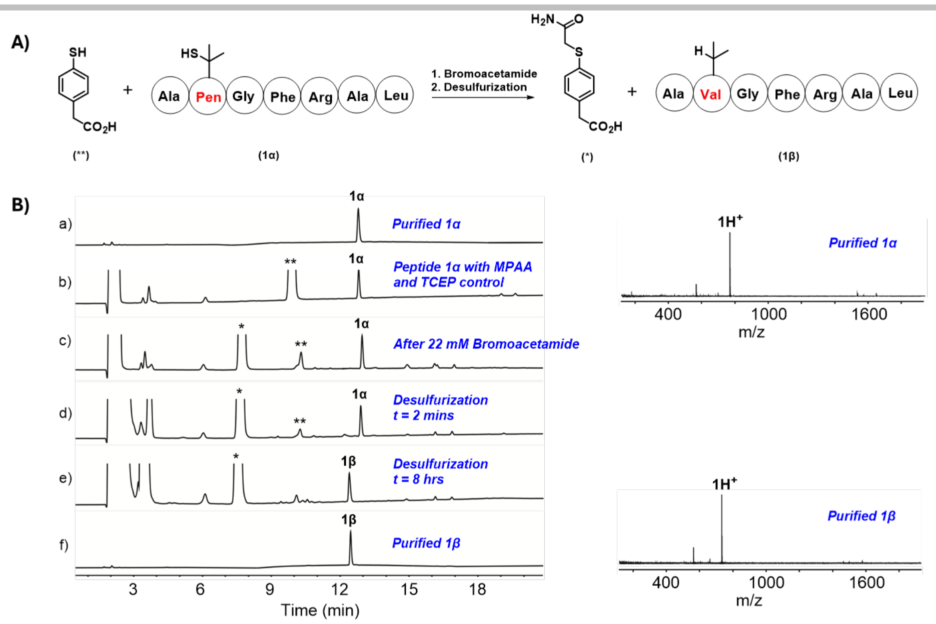

Figure S13. (A) Reaction schematic. (B) Analytical RP-HPLC profile (λ = 214 nm) and ESI-MS data (right) for the reaction of MPAA (**) with bromoacetamide in the presence of N-acetylcysteine and peptide **1α**. Chromatogram of (a) the purified peptide Ala-Pen-Gly-Phe-Arg-Ala-Leu-$^{\alpha}$*CONH₂* (**1α**) (b) the reaction mixture containing peptide **1α**, 20 mM MPAA, 20 mM TCEP in the ligation buffer (6 M Gu.HCl, 0.2 M PB, pH 6.7) (c) the reaction mixture at 5 minutes after adding 100 mM N-acetylcysteine followed by 22 mM bromoacetamide (d) at t = 2 minutes after the initiation of desulfurization of peptide **1α** (e) desulfurized peptide **1β** after 8 hours (f) purified peptide **1β**. Linear gradient 01%-61% of B over 20 min including 4 min equilibration using Agilent Zorbax SB-C3 5 µm 4.6 x 150 mm, 120Å LC column with 0.9 mL/min flow rate was used for all the chromatographic separation. The '**' indicates MPAA and (*) indicates alkylated MPAA.

6. One-pot desulfurization of peptide *Ac*-Cys-Phe-Arg-Ala-Leu-$^{\alpha}$*COOH* (1) with ultrasound-induced desulfurization (USID) methodology

To show the compatibility of our one-pot ligation desulfurization strategy with the recently reported USID methodology[3] for the desulfurization of proteins, we used model peptide *Ac*-Cys-Phe-Arg-Ala-Leu-$^{\alpha}$*COOH* (1) for this study. To a solution of peptide **1** (1 mg, 1 mM), MPAA (5.15 mg, 20 mM), and TCEP (8.77 mg, 20 mM) dissolved in 1.54 mL of ligation buffer (200 mM PB, 6 M Gu.HCl) was added solid N-acetyl cysteine (25 mg, 100 mM) and the pH was adjusted to 6.6. Separately, bromoacetamide (4.65 mg, 22 mM) was dissolved in 50 µL of water and then added slowly (10 µL x 5) to the reaction mixture over 2 minutes. The reaction mixture was further stirred for an extra 2-3 minutes. To this solution (1.62 mL), solid TCEP (92.85 mg, 200 mM) was added, and the pH was readjusted to 7.4. Furthermore, TiO2 nanoparticles (0.5 mg/mL) were added to the reaction mixture. The reaction was kept in the ultrasound cleaning bath at 37°C until completion. After 7 hours, the desulfurized peptide (1a) was confirmed by RP-HPLC analysis (**Figure S14**) (Observed mass of **1a** (ESI-MS): 618.37 Da (deconvoluted most abundant isotopologue, monoisotopic); calculated mass of **1a**: 618.35 Da (monoisotopic).

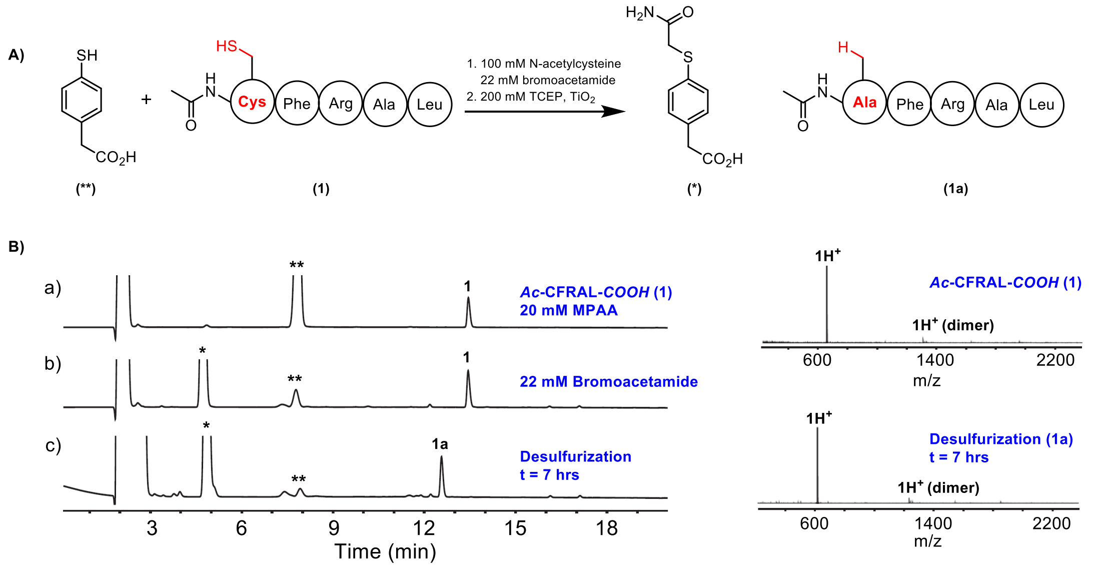

Figure S14. (A) Reaction schematic. (B) Analytical RP-HPLC profile (λ = 214 nm) and ESI-MS data (right) for the desulfurization of peptide **1** in the presence of MPAA (**) using USID strategy. Chromatogram of (a) the reaction mixture containing peptide *Ac*-Cys-Phe-Arg-Ala-Leu-$^{\alpha}$*COOH* (**1**) in the presence of 20 mM MPAA in the ligation buffer (6 M Gu.HCl, 0.2 M phosphate, 20 mM TCEP) and 100 mM N-acetylcysteine (b) the reaction mixture at 2 minutes after adding 22 mM bromoacetamide (c) desulfurized peptide **1a** after 7 hours. Linear gradient 10%-40% of B over 15 min including 4 min equilibration using Agilent Zorbax SB-C3 5 µm 4.6 x 150 mm, 300Å LC column with 0.9 mL/min flow rate was used for all the chromatographic separation. The '**' indicates MPAA and (*) indicates alkylated MPAA.

7. One-pot total chemical synthesis of Ubiquitin

7.1. Synthesis of ubiquitin peptide segment Met^{1}-Phe^{45}-$^{\alpha}$*COSR* (9)

The peptide with the sequence Met-Gln-Ile-Phe-Val-Lys-Thr-Leu-Thr-Gly-Lys-Thr-Ile-Thr-Leu-Glu-Val-Glu-Pro-Ser-Asp-Thr-Ile-Glu-Asn-Val-Lys-Ala-Lys-Ile-Gln-Asp-Lys-Glu-Gly-Ile-Pro-Pro-Asp-Gln-Gln-Arg-Leu-Ile-Phe-$^{\alpha}$*COSR* (**9**) was prepared from the corresponding peptide Met^{1}-Phe^{45}-Dbz-$^{\alpha}$*OH* segment **8** (**Figure S15a**). Stepwise synthesis of the peptide was carried out on Fmoc-[o-Boc]Dbz-Rink-Amide aminomethyl resin (substitution = 0.4 mmol/g) in a 0.1 mmol scale by machine-assisted SPPS at elevated temperature (see Section 1.3 for the peptide synthesis protocol). After the global deprotection of 0.04 mmol resin using a TFA cocktail, the crude peptide **8** was precipitated using cold diethyl ether. Observed mass (ESI-MS): 5247.82 Da (deconvoluted most abundant isotopologue); calculated mass: 5247.81 Da (most abundant isotopologue). The crude peptide **8** (200 mg) was then dissolved in 15 mL of aqueous phosphate buffer (0.2 M) containing 6 M Gu.HCl at pH 3.0 and kept at -16.5 °C (Julabo). After 15 min, 1.5 mL aqueous $NaNO_2$ (0.5 M) was added to the solution of peptide **8** and gently agitated for 15 min at -16.5 °C. Then, 15 mL of 0.3 M MESNa containing 0.2 M aqueous phosphate buffer and 6 M Gu.HCl at pH 6.3 was mixed into the oxidized solution of peptide **8** and the temperature was allowed to rise to RT. The MESNa exchange was complete within 30 min as observed from LCMS (**Figure S15b**). The pH of the resulting reaction mixture was adjusted to 3.8 and agitated further for 30 min. Purification of the crude reaction mixture by preparative HPLC gave 50.0 mg (09.54 µmol, 23.85% yield) of the desired MESNa exchanged peptide **9** (Met^{1}-Phe^{45}- $^{\alpha}$*COSR* **Figure S15c**). Observed mass (**ESI-MS**): 5237.78 Da (deconvoluted most abundant isotopologue); calculated mass: 5237.73 Da (most abundant isotopologue).

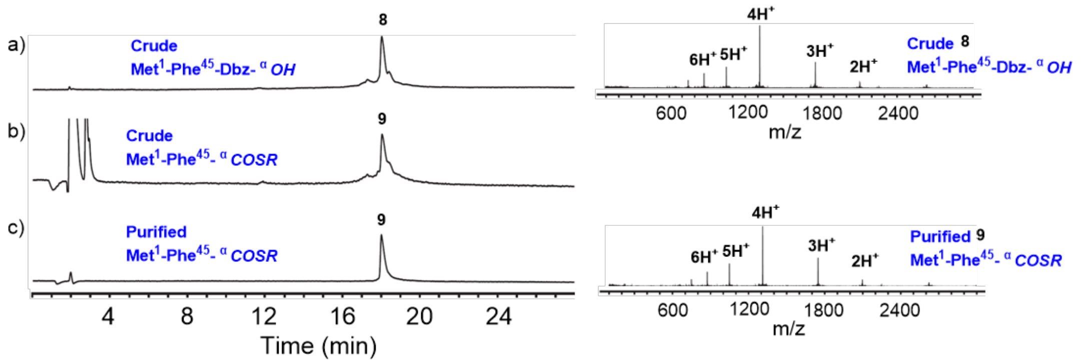

Figure S15. Analytical RP-HPLC profile (λ = 214 nm) together with ESI-MS data (right) for the synthesis of peptide Met^{1}-Phe^{45}- $^{\alpha}$*COSR* (**9**). (a) Chromatogram for the crude peptide Met^{1}-Phe^{45}-Dbz-$^{\alpha}$*OH* (**8**). (b) Chromatogram for the crude MESNa exchanged product Met^{1}-Phe^{45}- $^{\alpha}$*COSR* (**9**). (c) Chromatogram for the purified MESNa exchanged product Met^{1}-Phe^{45}- $^{\alpha}$*COSR* (**9**). $R = -CH_2CH_2SO_3Na$. Linear gradient 10%-54% of B over 22 min including 4 min equilibration using Agilent Zorbax SB-C3, 5µm, 4.6 x 150mm, 300Å LC column with 0.9 mL/min flow rate was used for all the chromatographic separation. Purification of the peptide was performed using a linear gradient of 10%-50% buffer B in buffer A over 80 min with a flow rate of 5 mL/min at 40 °C (buffer A = 0.1% TFA in water; buffer B = 0.08% TFA in acetonitrile) using a C3, 9.4 x 250 mm column (Agilent, 300 Å, 5 µm).

7.2. Synthesis of ubiquitin peptide segment Cys^{46}-Gly^{76}-$^{\alpha}$*COOH* (10)

The peptide Cys-Gly-Lys-Gln-Leu-Glu-Asp-Gly-Arg-Thr-Leu-Ser-Asp-Tyr-Asn-Ile-Gln-Lys-Glu-Ser-Thr-Leu-His-Leu-Val-Leu-Arg-Leu-Arg-Gly-Gly-$^{\alpha}$*COOH* (**10**) was obtained by machine-assisted SPPS at elevated temperature (see Section 1.3 for the peptide synthesis protocol). Stepwise synthesis of the peptide was carried out on 2-CTC-resin (substitution = 0.2 mmol/g) with a 0.15 mmol scale. Global deprotection and cleavage of the peptide from the resin using a TFA cocktail followed by HPLC purification gave 132.9 mg (37.96 µmol, 25% yield) of the desired peptide Cys^{46}-Gly^{76}-$^{\alpha}$*COOH* (**10**). Observed mass (ESI-MS): 3500.84 Da (deconvoluted most abundant isotopologue); calculated mass: 3499.83 Da (most abundant isotopologue) (**Figure S16**).

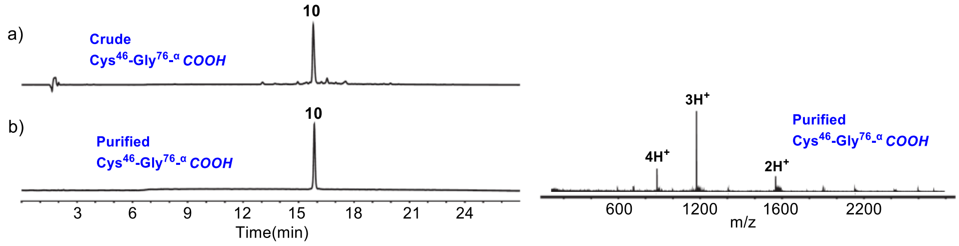

Figure S16. Analytical RP-HPLC profile (λ = 214 nm) together with ESI-MS data (right) for the synthesis of peptide Cys^{46}-Gly^{76}-$^{\alpha}$*COOH* (**10**). (a) Chromatogram for the crude peptide Cys^{46}-Gly^{76}-$^{\alpha}$*COOH* (**10**). (b) Chromatogram for the purified peptide Cys^{46}-Gly^{76}-$^{\alpha}$*COOH* (**10**). Linear gradient 10%-54% of B over 22 min including 4 min equilibration using Agilent Zorbax SB-C3, 5µm, 4.6 x 150mm LC column with 0.9 mL/min flow rate was used for all the chromatographic separation. Purification of the peptide was performed using a linear gradient of 15%-35% buffer B in buffer A over 60 min with a flow rate of 5 mL/min at 40 °C (buffer A = 0.1% TFA in water; buffer B = 0.08% TFA in acetonitrile) using a C4, 10 x 250 mm column (Phenomenex proteo, 300 Å, 10 µm).

7.3. Native chemical ligation of ubiquitin peptide segments 9 and 10 followed by one-pot desulfurization

To a solution of peptide **9** (5 mg, 0.95 µmol, 1 mM) in 954 µL of the ligation buffer (200 mM PB, 6 M Gu.HCl, 20 mM TCEP) containing 20 mM MPAA was added peptide **10** (3.34 mg, 0.95 µmol, 1 mM) and the pH of the reaction mixture was adjusted to 6.8. The ligation was completed in 6 h to yield the ligated product **11**. To this solution, N-acetylcysteine (15.5 mg, 95.0 µmol, 100 mM) was added solid, and the pH of the reaction mixture was measured and adjusted to 6.6. To this solution, bromoacetamide (2.63 mg, 19.08 µmol, 20 mM) dissolved in 20 µL water was added slowly over 2 minutes. The reaction mixture was stirred for an extra 2-3 minutes at room temperature. To this solution, TCEP (64.5 mg, 225.05 µmol, 150 mM), and VA-044 (48.4, 150 µmol, 100 mM) were added and the pH of the solution was adjusted to 7.0 and the reaction mixture was kept at 37°C until the completion of the reaction. The desulfurization was completed within 4 h, furnishing the full-length ubiquitin polypeptide **12.** The ligations and desulfurization were monitored by analytical HPLC and ESI-MS as shown in (main text **Figure 3-C(d), E,** and **Figure S17**). The purification of the full-length polypeptide by preparative HPLC afforded

6.0 mg (0.70 µmol, 73.6% yield based on the starting peptide segment **9**) of the pure desired peptide **12** (Observed mass (**ESI-MS**): 8564.67 Da (deconvoluted most abundant isotopologue); calculated mass: 8564.64 Da (most abundant isotopologue)).

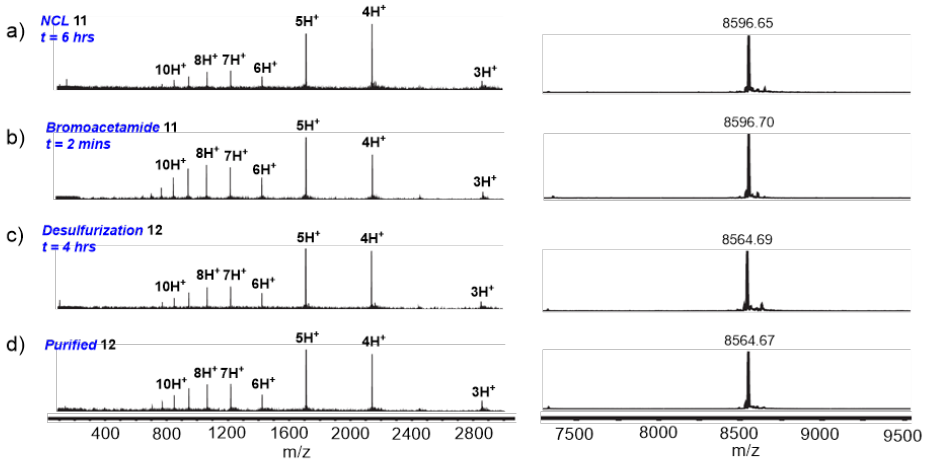

Figure S17. ESI-MS data (deconvoluted most abundant isotopologue on the right) of the ligation reaction followed by quenching of MPAA and one-pot desulfurization. ESI-MS data of the a) ligated polypeptide **11** after the ligation was over in 6 h (b) polypeptide **11** after 2 min of the addition of bromoacetamide (c) desulfurized polypeptide **12** after 4 h of the completion of desulfurization. RP-HPLC chromatogram data is shown in the main text *Figure 3-C*. Linear gradient 10%-54% of B over 22 min including 4 min equilibration using Agilent Zorbax SB-C3 5 µm 4.6 x 150 mm, 120Å LC column with 0.9 mL/min flow rate was used for all the chromatographic separation. Purification was performed using a linear gradient of 15%-45% buffer B in buffer A over 60 min with a flow rate of 5 mL/min at 40 °C (buffer A = 0.1% TFA in water; buffer B = 0.08% TFA in acetonitrile) using a C3, 9.4 x 250 mm column (Agilent, 300 Å, 5 µm).

7.4. Characterization of ubiquitin protein (12) by circular dichroism

The chemically synthesized full-length ubiquitin polypeptide was characterized by circular dichroism (CD) as mentioned in **section 1.5**. The sample for CD was prepared by dissolving lyophilized polypeptide **12** (0.2 mg) in 1 mL of 5 mM PB buffer at pH 7.4. The solution was filtered and kept at 4°C for 30 minutes before taking the spectra. The CD spectra is shown in **Figure 3-D**.

7.5. Synthesis of ubiquitin peptide segment Met^{1}-Phe^{45}- $^{\alpha}$*CONHNH$_2$ (13)*

The peptide Met-Gln-Ile-Phe-Val-Lys-Thr-Leu-Thr-Gly-Lys-Thr-Ile-Thr-Leu-Glu-Val-Glu-Pro-Ser-Asp-Thr-Ile-Glu-Asn-Val-Lys-Ala-Lys-Ile-Gln-Asp-Lys-Glu-Gly-Ile-Pro-Pro-Asp-Gln-Gln-Arg-Leu-Ile-Phe-$^{\alpha}$*CONHNH$_2$* (**13**) was synthesized using NH_2-NH-2-Cl-(Trt)-resin (scale = 0.05 mmol; substitution = 0.4 mmol/g) by stepwise Fmoc chemistry SPPS in an automated peptide synthesizer at 50 °C (*see* **Section-1.3** for the peptide synthesis protocol). After global deprotection using a TFA cocktail, crude peptide **13** was precipitated using cold diethyl ether. Purification using preparative HPLC gave 102 mg (19.89 µmol, 39.78% yield) of the pure peptide **13** (**Figure S18b**). Observed mass (ESI-MS): 5127.78 Da (deconvoluted most abundant isotopologue); calculated mass: 5127.79 Da (most abundant isotopologue).

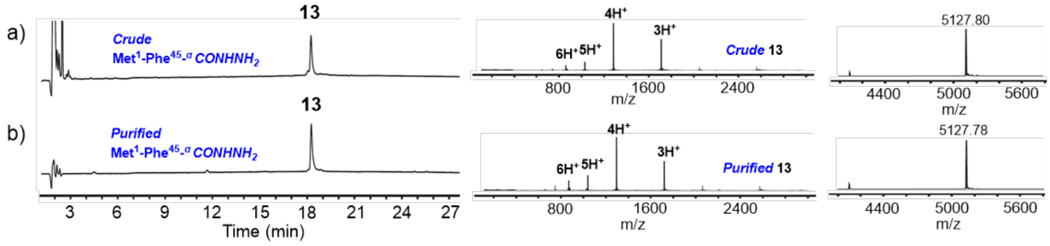

Figure S18. Analytical RP-HPLC profile (λ = 214 nm) together with ESI-MS data (right) for the synthesis of peptide Met^{1}-Phe^{45}- $^{\alpha}$*CONHNH$_2$ (13)*. (a) Chromatogram for the crude peptide Met^{1}-Phe^{45}- $^{\alpha}$*CONHNH$_2$ (13)*. (b) Chromatogram for the purified peptide Met^{1}-Phe^{45}- $^{\alpha}$*CONHNH$_2$ (13)*. Linear gradient 10%-54% of B over 22 min including 4 min equilibration using Agilent Zorbax SB-C3, 5µm, 4.6 x 150mm, 120Å LC column with 0.9 mL/min

flow rate was used for all the chromatographic separation. Purification of the peptide was performed using a linear gradient of 15%-35% buffer B in buffer A over 60 min with a flow rate of 5 mL/min at 40 °C (buffer A = 0.1% TFA in water; buffer B = 0.08% TFA in acetonitrile) using a C3, 9.4 x 250 mm column (Agilent, 300 Å, 5 µm).

7.6. Native chemical ligation of ubiquitin peptides 13 and 10 using Knorr pyrazole synthesis strategy to active hydrazide of segment 13 followed by one-pot desulfurization

To check the feasibility of one-pot ligation-desulfurization for Phil Dawson's hydrazide activation method,[4] we ligated ubiquitin hydrazide peptide segments **13** and **10** followed by one-pot desulfurization. To a solution of peptide **13** (5 mg, 0.97 µmol, 5 mM) in 195 µL of the ligation buffer (6 M Gu.HCl) containing a suspension of 200 mM MPAA (6.6 mg, 39.23 µmol) at pH 3.5 was added acetylacetone (acac) (0.25 µL, 2.43 µmol) and the reaction was stirred at RT until the completion of the reaction. The MPAA-thioester peptide **14** was formed after 10 h. To this solution, peptide **10** (3.34 mg, 0.95 µmol) dissolved in 400 µL of the ligation buffer (6 M Gu.HCl, 0.2 M PB, 50 mM TCEP, pH 9.0) was added and the pH was adjusted to 6.8. The ligation was completed within 4 h to yield the ligated polypeptide **11**. To this solution, N-acetylcysteine (9.8 mg, 60.05 µmol, 100 mM) was added solid, and the pH of the reaction mixture was measured and adjusted to 6.5. To this solution, bromoacetamide (6.59 mg, 39.23 µmol) dissolved in 40 µL water was added slowly over 2 minutes. The reaction mixture was stirred for an extra 2-3 minutes at room temperature. The solution was diluted to 975 µL to reach the peptide concentration of 1 mM and MPAA 40 mM. To this solution, TCEP (41.9 mg, 146.19 µmol, 150 mM), and VA-044 (31.5 mg, 97.5 µmol, 100 mM) were added and the pH of the solution was adjusted to 7.0 and the reaction mixture was kept at 37°C until the completion of the reaction. The desulfurization was completed within 4 h furnishing the full-length ubiquitin polypeptide **12**. The ligations and desulfurization were monitored by analytical HPLC and ESI-MS (main text **Figure 3-F(f), H,** and **Figure S19**). The purification of the full-length polypeptide by preparative HPLC afforded 5.65 mg (0.65 µmol, 67.0% yield based on the starting peptide segment **13**) of the pure desired peptide **12** (Observed mass (**ESI-MS**): 8564.66 Da (most abundant isotopologue); calculated mass: 8564.64 Da (most abundant isotopologue)).

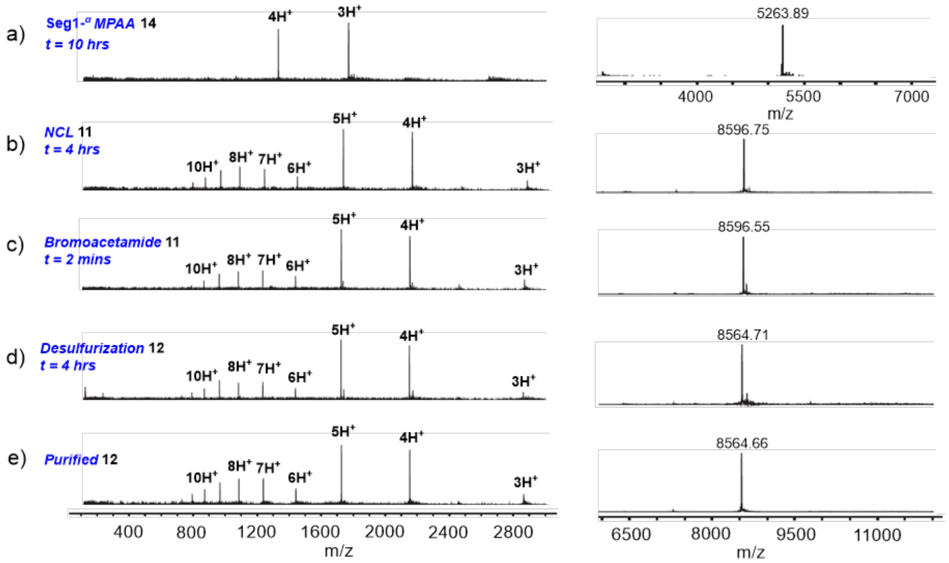

Figure S19. ESI-MS data of the ligation reaction followed by quenching of MPAA and one-pot desulfurization. ESI-MS data of the a) formation of Seg1-$^{\alpha}$*MPAA* (**14**) thioester formation after 10 h of the addition of acac (2.5 eq, 12.5 mM) and MPAA (200 mM) in the ligation buffer (6 M Gu.HCl) at pH 3.5. (b) ligated polypeptide **11** after the ligation was over in 4 h (c) polypeptide **11** after 2 min of the addition of bromoacetamide (d) desulfurized polypeptide **12** after 4 h of the completion of desulfurization. (e) purified desulfurized ubiquitin polypeptide **12**. RP-HPLC chromatogram data is shown in the main text *Figure 3-C*. Linear gradient 10%-54% of B over 22 min including 4 min equilibration using Agilent Zorbax SB-C3 5 µm 4.6 x 150 mm, 120Å LC column with 0.9 mL/min flow rate was used for all the chromatographic separation. Purification was performed using a linear gradient of 5%-25% buffer B in buffer A over 60 min with a flow rate of 5 mL/min at 40 °C (buffer A = 0.1% TFA in water; buffer B = 0.08% TFA in acetonitrile) using a C3, 9.4 x 250 mm column (Agilent, 300 Å, 5 µm).

7.7. Characterization of ubiquitin protein 12 by circular dichroism

The chemically synthesized full-length ubiquitin polypeptide (**12**) was characterized by circular dichroism as mentioned in section **1.5**. The sample for CD was prepared by dissolving lyophilized polypeptide 4 (0.2 mg) in 1 mL of 5 mM PB buffer at pH 7.4. The solution was filtered and kept at 4°C for 30 minutes before taking the spectra. The CD spectra is shown in **Figure 3-G**.

8. One-pot synthesis of collagen polypeptide Gly^{1}-Arg^{99}-$^{\alpha}$*COOH* (30) via five consecutive ligations followed by desulfurization

8.1. Synthesis of collagen peptide segment Gly^{1}-Lys^{20}- $^{\alpha}$*COSR* (16)

The peptide with the sequence Gly-Leu-Pro-Gly-Ala-Lys-Gly-Leu-Ala-Gly-Ala-Pro-Gly-Ala-Pro-Gly-Pro-Asp-Gly-Lys-$^{\alpha}$*COSR* (**16**) was prepared from the corresponding peptide hydrazide Gly^{1}-Lys^{20}-$^{\alpha}$*CONHNH$_2$* (**15**). The hydrazide peptide Gly^{1}-Lys^{20}-$^{\alpha}$*CONHNH$_2$* (**15**) was synthesized on NH_2NH-2-Cl-(Trt)-resin (substitution = 0.5 mmol/g) by stepwise Fmoc chemistry SPPS on a 0.1 mmol manually in a glass reaction vessel at RT (**see *Section-1.4*** for the peptide synthesis protocol). After the global deprotection using the TFA cocktail, the crude peptide **15** was precipitated using cold diethyl ether. The purification of the crude peptide by preparative HPLC afforded 93 mg (54.67 µmol, 54.67%) of the pure peptide **15** (**Figure S20b**). Observed mass (ESI-MS): 1700.94 Da (deconvoluted most abundant isotopologue); calculated mass: 1700.93 Da (most abundant isotopologue). The purified peptide **15** (40 mg, 23.51 µmol) was then dissolved in 10 mL of aqueous phosphate buffer (0.2 M) containing 6 M Gu.HCl at pH 3.0 and kept at -16.5 °C (Julabo). After 15 min, 1.0 mL aqueous $NaNO_2$ (0.5 M) was added to the solution of peptide **15** and gently agitated for 15 min at -16.5 °C. Then, 10 mL of 0.2 M MESNa containing 0.2 M aqueous phosphate buffer and 6 M Gu.HCl at pH 6.15 was mixed into the oxidized solution of peptide **15** and the temperature was allowed to rise to RT. The MESNa exchange was completed within 30 min as observed from LCMS (**Figure S20c**). The pH of the resulting reaction mixture was adjusted to 3.0. Purification of the crude reaction mixture by preparative HPLC gave 30.0 mg (16.56 µmol, 70.4% yield) of the desired MESNa exchanged peptide **16** (Gly^{1}-Lys^{20}- $^{\alpha}$*COSR* **Figure S20d**). Observed mass (**ESI-MS**): 1810.88 Da (deconvoluted most abundant isotopologue); calculated mass: 1810.87 Da (most abundant isotopologue).

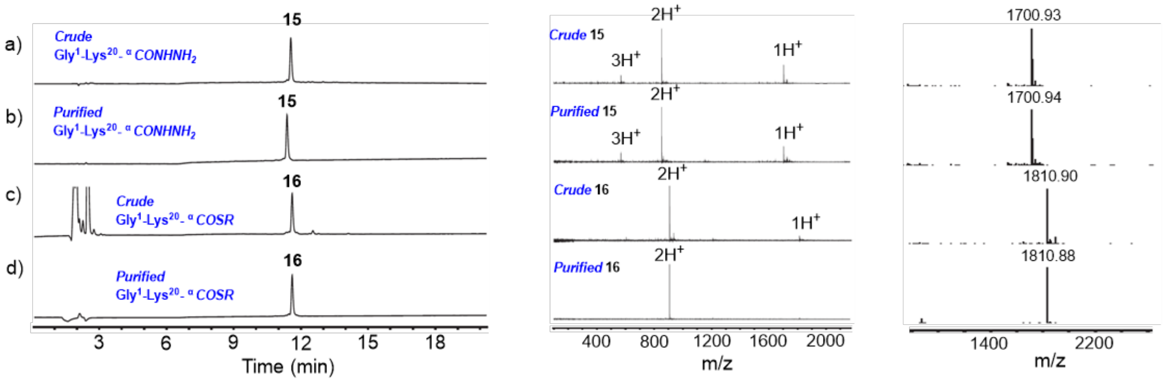

Figure S20. Analytical RP-HPLC profile (λ = 214 nm) together with ESI-MS data (right) for the synthesis of peptide Gly^{1}-Lys^{20}- $^{\alpha}$*COSR* (**16**). (a) Chromatogram for the crude peptide Gly^{1}-Lys^{20}- $^{\alpha}$*CONHNH$_2$* (**15**). (b) Chromatogram for the purified peptide Gly^{1}-Lys^{20}- $^{\alpha}$*CONHNH$_2$* (**15**). (c) Chromatogram for the crude MESNa exchanged product Gly^{1}-Lys^{20}-$^{\alpha}$*COSR* (**16**). (d) Chromatogram for the purified MESNa exchanged product Gly^{1}-Lys^{20}-$^{\alpha}$*COSR* (**16**). $R = -CH_2CH_2SO_3Na$. Linear gradient 10%-54% of B over 22 min including 4 min equilibration using Agilent Zorbax SB-C3, 5µm, 4.6 x 150mm, 120Å LC column with 0.9 mL/min flow rate was used for all the chromatographic separation. Purification of the peptide was performed using a linear gradient of 01%-61% buffer B in buffer A over 60 min with a flow rate of 5 mL/min at 40 °C (buffer A = 0.1% TFA in water; buffer B = 0.08% TFA in acetonitrile) using a C3, 9.4 x 250 mm column (Agilent, 300 Å, 5 µm).

8.2. Synthesis of collagen peptide segment *Fmoc*-Cys^{21}-Gly^{40}- $^{\alpha}$*COSR* (18)

The peptide with the sequence *Fmoc*-Cys-Gly-Pro-Pro-Gly-Pro-Ala-Gly-Gln-Asp-Gly-Arg-Pro-Gly-Pro-Pro-Gly-Pro-Pro-Gly-$^{\alpha}$*COSR* (**18**) was prepared from the corresponding peptide hydrazide *Fmoc*-Cys^{21}-Gly^{40}- $^{\alpha}$*CONHNH$_2$* (**17**). The hydrazide peptide *Fmoc*-Cys^{21}-Gly^{40}-$^{\alpha}$*CONHNH$_2$* (**17**) was synthesized on NH_2NH-2-Cl-(Trt)-resin (substitution = 0.7 mmol/g) by stepwise Fmoc chemistry SPPS on a 0.1 mmol scale manually in a glass reaction vessel at RT (**see *Section-1.4*** for the peptide synthesis protocol). After the global deprotection using the TFA cocktail, the crude peptide **17** was precipitated using cold diethyl ether. The purification of the crude peptide by preparative HPLC afforded 95 mg (47.40 µmol, 47.4%) of the pure peptide **17** (**Figure S21b**). Observed mass (ESI-MS): 2003.90 Da (deconvoluted most abundant isotopologue); calculated mass: 2003.91 Da (most abundant isotopologue). The purified peptide **17** (20 mg, 09.98 µmol) was then dissolved in 5 mL of aqueous phosphate buffer (0.2 M) containing 6 M Gu.HCl at pH 3.0 and kept at -16.5 °C (Julabo). After 15 min, 0.5 mL aqueous $NaNO_2$ (0.5 M) was added to the solution of peptide **17** and gently agitated for 15 min at -16.5 °C. Then, 5 mL of 0.2 M MESNa

containing 0.2 M aqueous phosphate buffer and 6 M Gu.HCl at pH 6.25 was mixed into the oxidized solution of peptide **17** and the temperature was allowed to rise to RT. The MESNa exchange was completed within 30 min as observed from LCMS (**Figure S21c**). Afterward, 150 mg of TCEP (50 mM) was added to the reaction mixture as a solid powder. The pH of the resulting reaction mixture was adjusted to 3.5 and agitated further for 30 min. Purification of the crude reaction mixture by preparative HPLC gave 14.0 mg (06.62 μmol, 66.33% yield) of the desired MESNa exchanged peptide **18** (*Fmoc*-Cys^{21}-Gly^{40}- $^{\alpha}$*COSR* **Figure S21d**). Observed mass (**ESI-MS**): 2113.88 Da (deconvoluted most abundant isotopologue); calculated mass: 2113.85 Da (most abundant isotopologue).

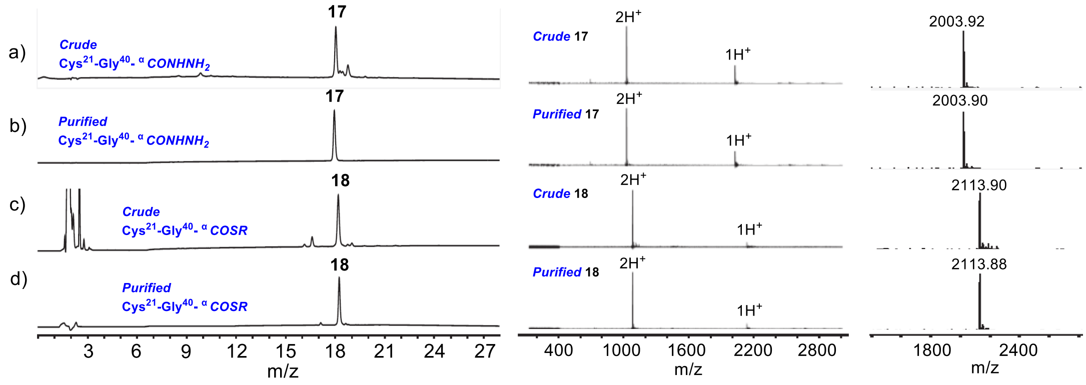

Figure S21. Analytical RP-HPLC profile (λ = 214 nm) together with ESI-MS data (right) for the synthesis of peptide *Fmoc*-Cys^{21}-Gly^{40}- $^{\alpha}$*COSR* (**18**). (a) Chromatogram for the crude peptide *Fmoc*-Cys^{21}-Gly^{40}- $^{\alpha}$*CONHNH$_2$* (**17**). (b) Chromatogram for the purified peptide *Fmoc*-Cys^{21}-Gly^{40}- $^{\alpha}$*CONHNH$_2$* (**17**). (c) Chromatogram for the crude MESNa exchanged product *Fmoc*-Cys^{21}-Gly^{40}- $^{\alpha}$*COSR* (**18**). (d) Chromatogram for the purified MESNa exchanged product *Fmoc*-Cys^{21}-Gly^{40}- $^{\alpha}$*COSR* (**18**). *R = –CH$_2$CH$_2$SO$_3$Na.* Linear gradient 10%-54% of B over 22 min including 4 min equilibration using Agilent Zorbax SB-C3, 5μm, 4.6 x 150mm, 120Å LC column with 0.9 mL/min flow rate was used for all the chromatographic separation. Purification of the peptide was performed using a linear gradient of 15%-35% buffer B in buffer A over 60 min with a flow rate of 5 mL/min at 40 °C (buffer A = 0.1% TFA in water; buffer B = 0.08% TFA in acetonitrile) using a C3, 9.4 x 250 mm column (Agilent, 300 Å, 5 μm).

8.3. Synthesis of collagen peptide segment *Fmoc*-Cys^{41}-Ala^{56}- $^{\alpha}$*COSR* (20)

The peptide with the sequence *Fmoc*-Cys-Arg-Gly-Gln-Ala-Gly-Pro-Pro-Gly-Phe-Pro-Gly-Pro-Lys-Gly-Ala-$^{\alpha}$*COSR* (**20**) was prepared from the corresponding peptide hydrazide *Fmoc*-Cys^{41}-Ala^{56}- $^{\alpha}$*CONHNH$_2$* (**19**). The hydrazide peptide *Fmoc*-Cys^{41}-Ala^{56}- $^{\alpha}$*CONHNH$_2$* (**19**) was synthesized on NH$_2$NH-2-Cl-(Trt)-resin (substitution = 0.5 mmol/g) by stepwise Fmoc chemistry SPPS on a 0.1 mmol manually in a glass reaction vessel at RT (**see *Section-1.4*** for the peptide synthesis protocol). After the global deprotection using the TFA cocktail, the crude peptide **19** was precipitated using cold diethyl ether. The purification of the crude peptide by preparative HPLC afforded 90 mg (51.96 μmol, 51.96 %) of the pure peptide **19** (**Figure S22b**). Observed mass (ESI-MS): 1731.84 Da (deconvoluted most abundant isotopologue); calculated mass: 1731.83 Da (most abundant isotopologue). The purified peptide **19** (40 mg, 23.08 μmol) was then dissolved in 10 mL of aqueous phosphate buffer (0.2 M) containing 6 M Gu.HCl at pH 3.0 and kept at -16.5 °C (Julabo). After 15 min, 1.0 mL aqueous $NaNO_2$ (0.5 M) was added to the solution of peptide **19** and gently agitated for 15 min at -16.5 °C. Then, 10 mL of 0.2 M MESNa containing 0.2 M aqueous phosphate buffer and 6 M Gu.HCl at pH 6.15 was mixed into the oxidized solution of peptide **19** and the temperature was allowed to rise to RT. The MESNa exchange was completed within 30 min as observed from LCMS (**Figure S22c**). Afterward, 300 mg of TCEP (50 mM) was added to the reaction mixture as a solid powder. The pH of the resulting reaction mixture was adjusted to 3.8 and agitated further for 30 min. Purification of the crude reaction mixture by preparative HPLC gave 28.6 mg (15.52 μmol, 67.2% yield) of the desired MESNa exchanged peptide **20** (*Fmoc*-Cys^{41}-Ala^{56}- $^{\alpha}$*COSR* **Figure S22d**). Observed mass (**ESI-MS**): 1842.79 Da (deconvoluted most abundant isotopologue); calculated mass: 1842.77 Da (most abundant isotopologue).

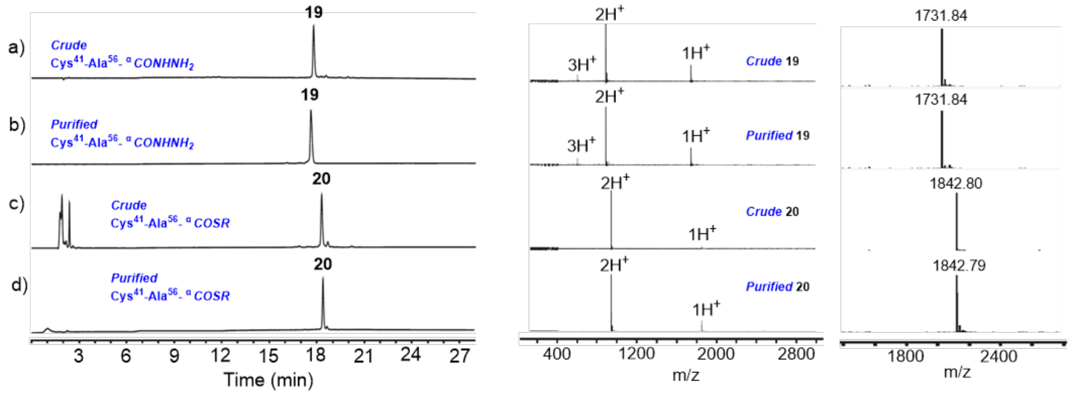

Figure S22. Analytical RP-HPLC profile (λ = 214 nm) together with ESI-MS data (right) for the synthesis of peptide *Fmoc*-Cys^{41}-Ala^{56}- $^{\alpha}$*COSR* (**20**). (a) Chromatogram for the crude peptide *Fmoc*-Cys^{41}-Ala^{56}-$^{\alpha}$*CONHNH$_2$* (**19**). (b) Chromatogram for the purified peptide *Fmoc*-Cys^{41}-Ala^{56}-$^{\alpha}$*CONHNH$_2$* (**19**). (c) Chromatogram for the crude MESNa exchanged product *Fmoc*-Cys^{41}-Ala^{56}- $^{\alpha}$*COSR* (**20**). (d) Chromatogram for the purified MESNa exchanged product *Fmoc*-Cys^{41}-Ala^{56}- $^{\alpha}$*COSR* (**20**). *R = –CH$_2$CH$_2$SO$_3$Na.* Linear gradient 10%-54% of B over 22 min including 4 min equilibration using Agilent Zorbax SB-C3, 5µm, 4.6 x 150mm, 120Å LC column with 0.9 mL/min flow rate was used for all the chromatographic separation. Purification of the peptide was performed using a linear gradient of 15%-35% buffer B in buffer A over 60 min with a flow rate of 5 mL/min at 40 °C (buffer A = 0.1% TFA in water; buffer B = 0.08% TFA in acetonitrile) using a C3, 9.4 x 250 mm column (Agilent, 300 Å, 5 µm).

8.4. Synthesis of collagen peptide segment *Fmoc*-Cys^{57}-Gly^{73}- $^{\alpha}$*COSR* (22)

The peptide with the sequence *Fmoc*-Cys-Gly-Glu-Pro-Gly-Lys-Ala-Gly-Glu-Arg-Gly-Val-Pro-Gly-Pro-Pro-Gly-$^{\alpha}$*COSR* (**22**) was prepared from the corresponding peptide hydrazide *Fmoc*-Cys^{57}-Gly^{73}- $^{\alpha}$*CONHNH$_2$* (**21**). The hydrazide peptide *Fmoc*-Cys^{57}-Gly^{73}- $^{\alpha}$*CONHNH$_2$* (**21**) was synthesized on NH_2NH-2-Cl-(Trt)-resin (substitution = 0.7 mmol/g) by stepwise Fmoc chemistry SPPS on a 0.1 mmol manually in a glass reaction vessel at RT (**see *Section-1.4*** for the peptide synthesis protocol). After the global deprotection using TFA cocktail, the crude peptide **21** was precipitated using cold diethyl ether. The purification of the crude peptide by preparative HPLC afforded 99.5 mg (55.25 µmol, 55.2%) of the pure peptide **21** (**Figure S23b**). Observed mass (ESI-MS): 1800.90 Da (deconvoluted most abundant isotopologue); calculated mass: 1800.88 Da (most abundant isotopologue). The purified peptide **21** (40 mg, 22.21 µmol) was then dissolved in 10 mL of aqueous phosphate buffer (0.2 M) containing 6 M Gu.HCl at pH 3.0 and kept at -16.5 °C (Julabo). After 15 min, 1.0 mL aqueous $NaNO_2$ (0.5 M) was added to the solution of peptide **21** and gently agitated for 15 min at -16.5 °C. Then, 10 mL of 0.2 M MESNa containing 0.2 M aqueous phosphate buffer and 6 M Gu.HCl at pH 6.3 was mixed into the oxidized solution of peptide **21** and the temperature was allowed to rise to RT. The MESNa exchange was completed within 30 min as observed from LCMS (**Figure S23c**). Afterward, 300 mg of TCEP (50 mM) was added to the reaction mixture as a solid powder. The pH of the resulting reaction mixture was adjusted to 4.0 and agitated further for 30 min. Purification of the crude reaction mixture by preparative HPLC gave 30.5 mg (15.96 µmol, 71.8% yield) of the desired MESNa exchanged peptide **22** (*Fmoc*-Cys^{57}-Gly^{73}- $^{\alpha}$*COSR* **Figure S23d**). Observed mass (**ESI-MS**): 1910.78 Da (deconvoluted most abundant isotopologue); calculated mass: 1910.78 Da (most abundant isotopologue).

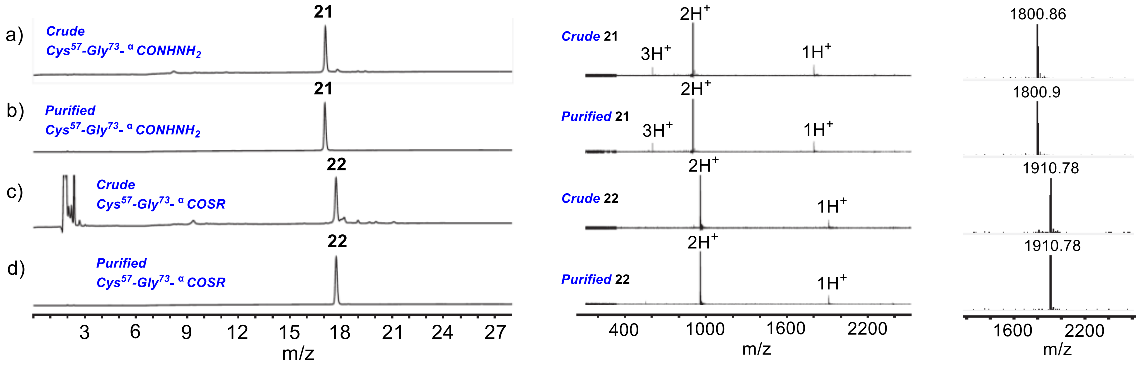

Figure S23. Analytical RP-HPLC profile (λ = 214 nm) together with ESI-MS data (right) for the synthesis of peptide *Fmoc*-Cys^{57}-Gly^{73}- $^{\alpha}$*COSR* (**22**). (a) Chromatogram for the crude peptide *Fmoc*-Cys^{57}-Gly^{73}-$^{\alpha}$*CONHNH$_2$* (**21**). (b) Chromatogram for the purified peptide *Fmoc*-Cys^{57}-Gly^{73}-$^{\alpha}$*CONHNH$_2$* (**21**). (c) Chromatogram for the crude MESNa exchanged product *Fmoc*-Cys^{57}-Gly^{73}- $^{\alpha}$*COSR* (**22**). (d) Chromatogram for the purified MESNa

exchanged product *Fmoc*-Cys[57]-Gly[73]- $^{\alpha}$*COSR* (**22**). *R = –CH₂CH₂SO₃Na.* Linear gradient 10%-54% of B over 22 min including 4 min equilibration using Agilent Zorbax SB-C3, 5µm, 4.6 x 150mm, 120Å LC column with 0.9 mL/min flow rate was used for all the chromatographic separation. Purification of the peptide was performed using a linear gradient of 15%-35% buffer B in buffer A over 60 min with a flow rate of 5 mL/min at 40 °C (buffer A = 0.1% TFA in water; buffer B = 0.08% TFA in acetonitrile) using a C3, 9.4 x 250 mm column (Agilent, 300 Å, 5 µm).

8.5. Synthesis of collagen peptide segment *Fmoc*-Cys^{74}-Gly^{85}- $^{\alpha}$*COSR* (24)

The peptide with the sequence *Fmoc*-Cys-Val-Gly-Pro-Ala-Gly-Lys-Asp-Gly-Glu-Ala-Gly-$^{\alpha}$*COSR* (**24**) was prepared from the corresponding peptide hydrazide *Fmoc*-Cys^{74}-Gly^{85}- $^{\alpha}$*CONHNH₂* (**23**). The hydrazide peptide *Fmoc*-Cys^{74}-Gly^{85}- $^{\alpha}$*CONHNH₂* (**23**) was synthesized on NH_2NH-2-Cl-(Trt)-resin (substitution = 0.7 mmol/g) by stepwise Fmoc chemistry SPPS on a 0.1 mmol manually in a glass reaction vessel at RT (**see *Section-1.4*** for the peptide synthesis protocol). After the global deprotection using the TFA cocktail, the crude peptide **23** was precipitated using cold diethyl ether. The purification of the crude peptide by preparative HPLC afforded 78 mg (60.20 µmol, 60.2%) of the pure peptide **23** (**Figure S24b**). Observed mass (ESI-MS): 1295.56 Da (deconvoluted most abundant isotopologue); calculated mass: 1295.56 Da (most abundant isotopologue). The purified peptide **23** (40 mg, 30.8 µmol) was then dissolved in 10 mL of aqueous phosphate buffer (0.2 M) containing 6 M Gu.HCl at pH 3.0 and kept at -16.5 °C (Julabo). After 15 min, 1.0 mL aqueous $NaNO_2$ (0.5 M) was added to the solution of peptide **23** and gently agitated for 15 min at -16.5 °C. Then, 10 mL of 0.2 M MESNa containing 0.2 M aqueous phosphate buffer and 6 M Gdn.HCl at pH 6.5 was mixed into the oxidized solution of peptide **23** and the temperature was allowed to rise to RT. The MESNa exchange was complete within 30 min as observed from LCMS (**Figure S24c**). Afterward, 300 mg of TCEP (50 mM) was added to the reaction mixture as a solid powder. The pH of the resulting reaction mixture was adjusted to 3.7 and agitated further for 30 min. Purification of the crude reaction mixture by preparative HPLC gave 25.5 mg (18.14 µmol, 58.89% yield) of the desired MESNa exchanged peptide **24** (*Fmoc*-Cys^{74}-Gly^{84}- $^{\alpha}$*COSR* **Figure S24d**). Observed mass (**ESI-MS**): 1405.51 Da (deconvoluted most abundant isotopologue); calculated mass: 1405.49 Da (most abundant isotopologue).

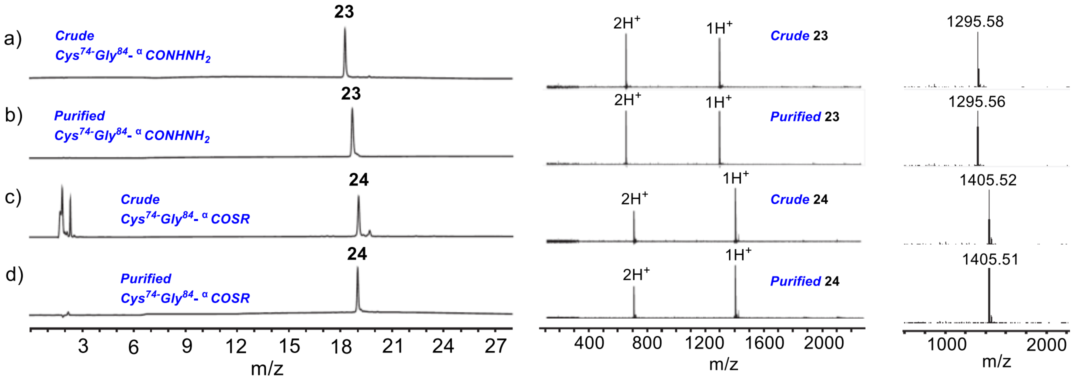

Figure S24. Analytical RP-HPLC profile (λ = 214 nm) together with ESI-MS data (right) for the synthesis of peptide *Fmoc*-Cys^{74}-Gly^{84}- $^{\alpha}$*COSR* (**24**). (a) Chromatogram for the crude peptide *Fmoc*-Cys^{74}-Gly^{85}-$^{\alpha}$*CONHNH₂* (**23**). (b) Chromatogram for the purified peptide *Fmoc*-Cys^{74}-Gly^{85}-$^{\alpha}$*CONHNH₂* (**23**). (c) Chromatogram for the crude MESNa exchanged product *Fmoc*-Cys^{74}-Gly^{85}- $^{\alpha}$*COSR* (**24**). (d) Chromatogram for the purified MESNa exchanged product *Fmoc*-Cys^{74}-Gly^{85}- $^{\alpha}$*COSR* (**24**). *R = –CH₂CH₂SO₃Na.* Linear gradient 10%-54% of B over 22 min including 4 min equilibration using Agilent Zorbax SB-C3, 5µm, 4.6 x 150mm, 120Å LC column with 0.9 mL/min flow rate was used for all the chromatographic separation. Purification of the peptide was performed using a linear gradient of 15%-35% buffer B in buffer A over 60 min with a flow rate of 5 mL/min at 40 °C (buffer A = 0.1% TFA in water; buffer B = 0.08% TFA in acetonitrile) using a C3, 9.4 x 250 mm column (Agilent, 300 Å, 5 µm).

8.6. Synthesis of collagen peptide segment Cys^{86}-Arg^{99}- $^{\alpha}$*COOH* (25)

The peptide Cys-Gln-Gly-Pro-Pro-Gly-Pro-Ala-Gly-Pro-Ala-Gly-Glu-Arg-$^{\alpha}$*COOH* (**25**) was synthesized on 2-Cl-(Trt)-resin (substitution= 0.5 mmol/g) by stepwise Fmoc chemistry SPPS on a 0.1 mmol scale manually in a glass reaction vessel at RT (**see *Section-1.4*** for the peptide synthesis protocol). Global deprotection and the cleavage of the peptide from the resin using a TFA cocktail followed by HPLC purification afforded 86 mg (66.53 µmol, 66.53 %) of the desired peptide **25,** Cys^{86}-Arg^{99}-$^{\alpha}$*COOH* (**Figure S25**). Observed mass (**ESI-MS**): 1292.62 Da (deconvoluted most abundant isotopologue); calculated mass: 1292.60 Da (most abundant isotopologue).

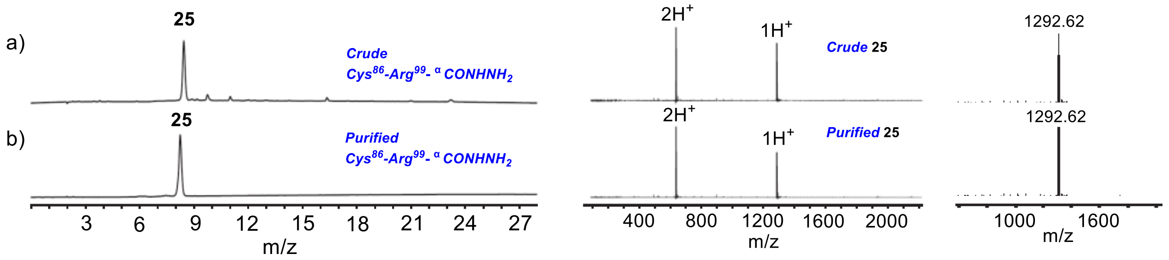

Figure S25. Analytical RP-HPLC profile (λ = 214 nm) together with ESI-MS data (right) for the synthesis of peptide Cys^{86}-Arg^{99}- $^{\alpha}$*COOH* (**25**). (a) Chromatogram for the crude peptide Cys^{86}-Arg^{99}- $^{\alpha}$*COOH* (**25**). (b) Chromatogram for the purified peptide Cys^{86}-Arg^{99}- $^{\alpha}$*COOH* (**25**). Linear gradient 10%-54% of B over 22 min including 4 min equilibration using Agilent Zorbax SB-C3, 5µm, 4.6 x 150mm, 120Å LC column with 0.9 mL/min flow rate was used for all the chromatographic separation. Purification of the peptide was performed using a linear gradient of 15%-35% buffer B in buffer A over 60 min with a flow rate of 5 mL/min at 40 °C (buffer A = 0.1% TFA in water; buffer B = 0.08% TFA in acetonitrile) using a C3, 9.4 x 250 mm column (Agilent zorbax, 300 Å, 5 µm).

8.7. One-pot six-segment ligation of collagen peptide segments 16, 18, 20, 22, 24, and 25 followed by one-pot desulfurization

All the six segments were ligated in a one-pot manner using the reported protocol.[5] To a solution of peptide **24** (2.00 mg, 1.42 µmol) in 178 µL of the ligation buffer (200 mM PB, 6 M Gu.HCl, pH 7.0) containing 80 mM MPAA (2.4 mg, 14.2 µmol), was added peptide **25** (1.83 mg, 1.42 µmol) and the pH of the reaction mixture was adjusted to 6.8. The first ligation was completed in 2 h to yield the ligated product **24'**. Then, piperidine was added to the reaction mixture, and the pH was adjusted to 11.0 by adding concentrated HCl maintaining the final concentration of piperidine to 20% (v/v) to deprotect the Fmoc group from the ligated product **24'** to yield peptide **26**. Immediately after 7 min of incubation, the pH of the reaction mixture was rapidly reduced to 9.0 by adding concentrated HCl. Solid TCEP (4.1 mg, ~80 mM) was then added, and the pH was readjusted to 7.0. The third peptide segment **22** (2.70 mg, 1.42 µmol) was then added to the reaction mixture, and the pH was readjusted to 6.85. The second ligation reaction was completed within 2 h. To yield the Fmoc deprotected peptide **27** from the ligated product **22'**, the pH was raised to 11.0 by adding 6M NaOH maintaining the final piperidine concentration to 20% (v/v). After 7 min, the pH of the reaction mixture was rapidly reduced to 7.0 by adding concentrated HCl followed by solid TCEP (5.7 mg, ~80 mM for 0.25 mL). After 5 min, the fourth peptide segment **20** (2.62 mg, 1.42 µmol) was added to the same reaction mixture and the pH was readjusted to 6.85. The third ligation was completed within 6 h furnishing the ligated polypeptide **20'**. To yield the Fmoc deprotected peptide **28** from the ligated product **20'**, the pH was raised to 11.0 by adding 6M NaOH and a small amount of piperidine maintaining the final piperidine concentration to 20% (v/v). After 7 min, the pH of the reaction mixture was rapidly reduced to 7.0 by adding concentrated HCl followed by solid TCEP (5.0 mg, ~50 mM for 0.35 mL). After 5 min, the fifth peptide segment **18** (3.0 mg, 1.42 µmol) was added to the same reaction mixture, and the pH was readjusted to 6.8. The fourth ligation was completed within 3 h furnishing the ligated polypeptide **18'**. To yield the Fmoc deprotected peptide **29** from the ligated product **18'**, the pH was raised to 11.0 by adding 6 M NaOH and maintaining the final piperidine concentration to 20% (v/v). After 7 min, the pH of the reaction mixture was rapidly reduced to 7.0 by adding concentrated HCl followed by solid TCEP (6.4 mg, ~50 mM for 0.45 mL). After 5 min, the sixth and last peptide segment **16** (2.57 mg, 1.42 µmol) was added to the same reaction mixture, and the pH was readjusted to 6.85. The fifth ligation was completed within 5 h furnishing the ligated polypeptide **16'**. To this solution, N-acetylcysteine (9.80 mg, 60.05 µmol, 100 mM for 0.6 mL) was added as solid, and the pH of the reaction mixture was adjusted to 6.5. To this solution, bromoacetamide (1.95 mg, 14.20 µmol) dissolved in 40 µL water was added slowly over 2 minutes. The reaction mixture was stirred for an extra 2-3 minutes at room temperature (trace amount of MPAA was left unreacted). To this solution, TCEP (34.4 mg, 120.12 µmol, 150 mM for 0.8 mL), and VA-044 (25.8 mg, 79.87 µmol, 100 mM for 0.8 mL) were added and the pH of the solution was adjusted to 7.0. The reaction mixture was kept at 37°C until the completion of the reaction. The desulfurization was completed within 8 h furnishing the full-length collagen polypeptide **30**. The one-pot ligations and desulfurization were monitored by analytical HPLC and ESI-MS (main text **Figure 4-C, D, E,** and **Figure S26**). The purification of the full-length polypeptide by preparative HPLC afforded 5.25 mg (0.61 µmol, 42.95% yield based on the starting peptide segment **24**) of the pure desired peptide **30** (Observed mass (**ESI-MS**): 8619.42 Da (deconvoluted most abundant isotopologue); calculated mass: 8619.35 Da (most abundant isotopologue))

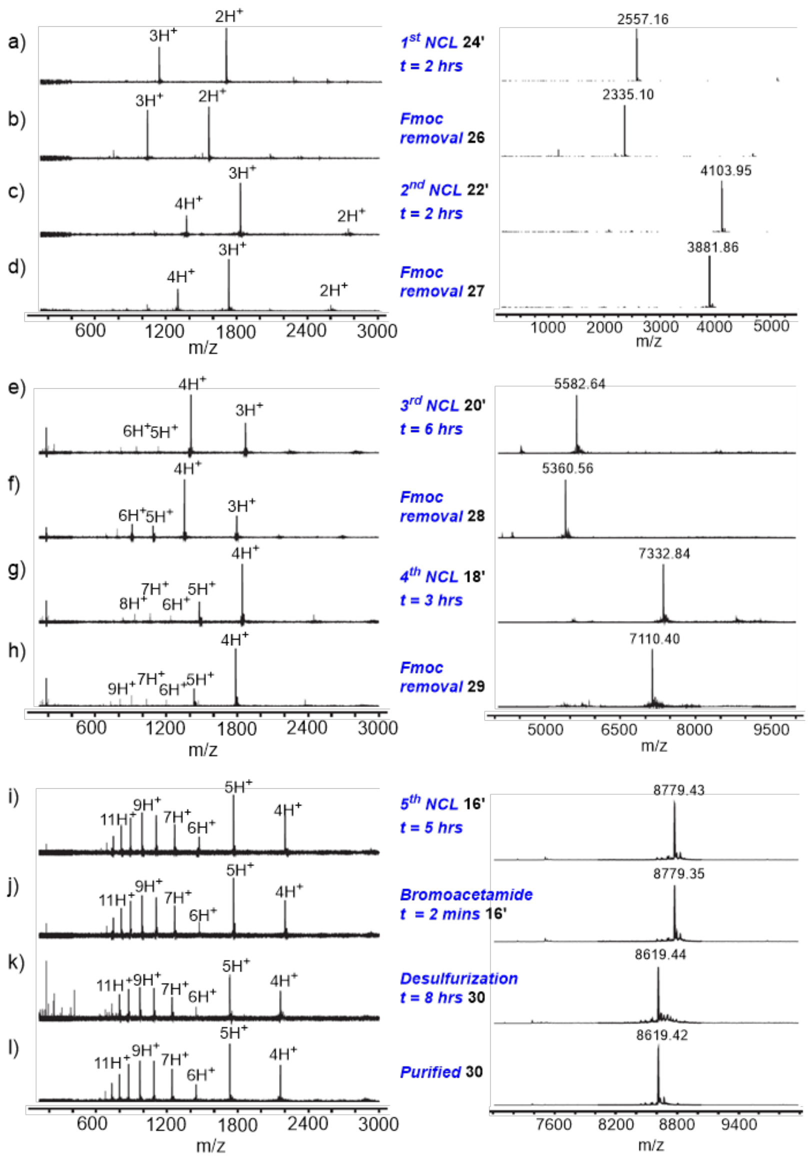

Figure S26. ESI-MS data of the one-pot synthesis of the collagen polypeptide. ESI-MS data (a) after the first ligation was completed within 2 h and gave **24'** as the ligated product (b) after Fmoc deprotection from the ligated product **24'** to obtain peptide **26**; (c) after the second ligation was completed within 2 h and gave **22'** as the ligated product; (d) after Fmoc deprotection from the ligated product **22'** to obtain peptide **27**; (e) after the third ligation was completed within 6 h and gave **20'** as the ligated product; (f) after Fmoc deprotection from the ligated product **20'** to obtain peptide **28**; (g) after the fourth ligation was completed within 3 h and gave **18'** as the ligated product; (h) after Fmoc deprotection from the ligated product **18'** to obtain peptide **29**; (i) after the fifth ligation was completed within 5 h and gave **16'** as the ligated product; (j) after 2 min of the addition of bromoacetamide in the presence of acetylcysteine to peptide **16'**; (k) after 8 h of desulfurization of peptide **16'** to give peptide **30**; (l) of the full-length purified collagen polypeptide **30.** Purification was carried out using a C3, 9.4 x 250 mm column (Agilent zorbax, 300 Å, 5 µm) with a linear gradient of 01%-41% of buffer B over 40 min with a flow rate of 5 mL/min at 40 °C.

9. One-pot total chemical synthesis of barstar A protein

9.1. Synthesis of barstar A segment Lys^{1}-Leu^{24} - $^{\alpha}$*COSR* (32)

The peptide Lys-Lys-Ala-Val-Ile-Asn-Gly-Glu-Gln-Ile-Arg-Ser-Ile-Ser-Asp-Leu-His-Gln-Thr-Leu-Lys-Lys-Glu-Leu-$^{\alpha}$*COSR* (**32**) was prepared from the corresponding Dbz peptide Lys^{1}-Leu^{24}-Dbz-$^{\alpha}$*OH* **31**. Stepwise synthesis of the peptide was carried out on Fmoc-[o-Boc]Dbz-Rink-Amide aminomethyl resin (substitution = 0.4 mmol/g) in 0.1 mmol scale by machine-assisted SPPS at elevated temperature (see Section 1.3 for the peptide synthesis protocol). After the global deprotection of 0.05 mmol resin using a TFA cocktail, the crude peptide **31** was precipitated using cold diethyl ether. The crude peptide **31** (150 mg) was then dissolved in 15 mL of aqueous phosphate buffer (0.2 M) containing 6 M Gu.HCl at pH 3.0 and kept at -16.5 °C (Julabo). After 15 min, 1.5 mL aqueous $NaNO_2$ (0.5 M) was added to the

solution of peptide **31** and gently agitated for 15 min at -16.5 °C. Then, 15 mL of 0.3 M MESNa containing 0.2 M aqueous phosphate buffer and 6 M Gu.HCl at pH 6.3 was mixed into the oxidized solution of peptide **31** and the temperature was allowed to rise to RT. The pH of the reaction mixture was then adjusted to 6.2, and the MESNa exchange was complete within 30 min as observed from LCMS (**Figure S27b**). Afterward, 430 mg of TCEP (50 mM) was added to the reaction mixture as a solid powder. The pH of the resulting reaction mixture was adjusted to 3.5 and agitated further for 30 min. Purification of the crude reaction mixture by preparative HPLC gave 77.75 mg (27.06 µmol, 54.12 % yield) of the desired MESNa exchanged peptide **32** (Lys^{1}-Leu^{24}- $^{\alpha}$*COSR,* **Figure S27c**). Observed mass (**ESI-MS**): 2872.55 Da (deconvoluted most abundant isotopologue); calculated mass: 2872.53 Da (most abundant isotopologue).

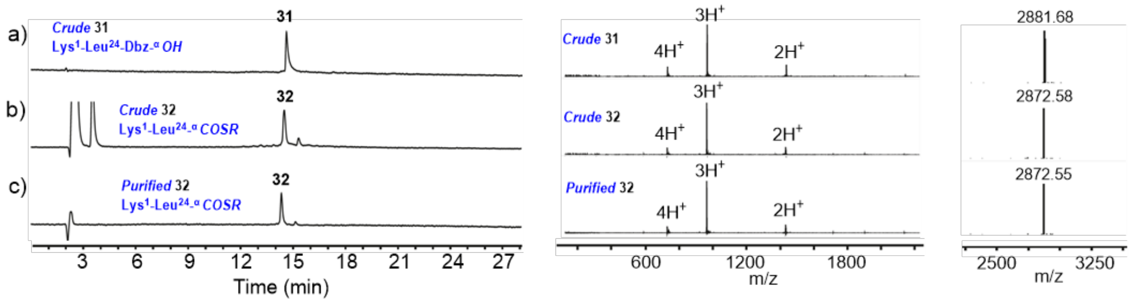

Figure S27. Analytical RP-HPLC profile (λ = 214 nm) together with ESI-MS data (right) for the synthesis of peptide Lys^{1}-Leu^{24}- $^{\alpha}$*COSR* (**32**). (a) Chromatogram for the crude peptide Lys^{1}-Leu^{24}-Dbz-$^{\alpha}$*OH* (**31**). (b) Chromatogram for the crude MESNa exchanged product Lys^{1}-Leu^{24}- $^{\alpha}$*COSR* (**32**). (c) Chromatogram for the purified MESNa exchanged product Lys^{1}-Leu^{24}- $^{\alpha}$*COSR* (**32**). *R* = –$CH_2CH_2SO_3Na$. Linear gradient 10%-54% of B over 22 min including 4 min equilibration using Agilent Zorbax SB-C3, 5µm, 4.6 x 150mm, 300Å LC column with 0.9 mL/min flow rate was used for all the chromatographic separation. Purification of the peptide was performed using a linear gradient of 05%-45% buffer B in buffer A over 80 min with a flow rate of 5 mL/min at 40 °C (buffer A = 0.1% TFA in water; buffer B = 0.08% TFA in acetonitrile) using a C3, 9.4 x 250 mm column (Agilent zorbax, 300 Å, 5 µm).

9.2. Synthesis of barstar A segment *Fmoc*-Cys^{25}-Gly^{66}- $^{\alpha}$*COSR* (34)

The peptide with the sequence *Fmoc*-Cys-Leu-Pro-Glu-Tyr-Tyr-Gly-Glu-Asn-Leu-Asp-Ala-Leu-Trp-Asp-Ala-Leu-Thr-Gly-Trp-Val-Glu-Tyr-Pro-Leu-Val-Leu-Glu-Trp-Arg-Gln-Phe-Glu-Gln-Ser-Lys-Gln-Leu-Thr-Glu-Asn-Gly-$^{\alpha}$*COSR* (**34**) was prepared from the corresponding Dbz peptide *Fmoc*-Cys^{25}-Gly^{66}-Dbz-$^{\alpha}$*OH* (**33**). Stepwise synthesis of the peptide was carried out on Fmoc-[o-Boc]Dbz-Rink-Amide aminomethyl resin (substitution = 0.4 mmol/g) in a 0.1 mmol scale by machine-assisted SPPS at elevated temperature (see Section 1.3 for the peptide synthesis protocol). After the global deprotection of 0.05 mmol resin using a TFA cocktail, the crude peptide **33** was precipitated using cold diethyl ether. The crude peptide **33** (215 mg) was then dissolved in 15 mL of aqueous phosphate buffer (0.2 M) containing 6 M Gu.HCl at pH 3.0 and kept at -16.5 °C (Julabo). After 15 min, 1.5 mL aqueous $NaNO_2$ (0.5 M) was added to the solution of peptide **33** and gently agitated for 15 min at -16.5 °C. Then, 15 mL of 0.3 M MESNa containing 0.2 M aqueous phosphate buffer and 6 M Gu.HCl at pH 6.3 was mixed into the oxidized solution of peptide **33** and the temperature was allowed to rise to RT. The pH of the reaction mixture was then adjusted to 6.2, and the MESNa exchange was complete within 30 min as observed from LCMS (**Figure S28b**). Afterward, 430 mg of TCEP (50 mM) was added to the reaction mixture as a solid powder. The pH of the resulting reaction mixture was adjusted to 3.8 and agitated further for 30 min. Purification of the crude reaction mixture by preparative HPLC gave 80.0 mg (14.94 µmol, 29.88% yield) of the desired MESNa exchanged peptide **34** (*Fmoc*-Cys^{25}-Gly^{66}- $^{\alpha}$*COSR* **Figure S28c**). Observed mass (**ESI-MS**):5251.52 Da (deconvoluted most abundant isotopologue); calculated mass: 5351.49 Da (most abundant isotopologue).

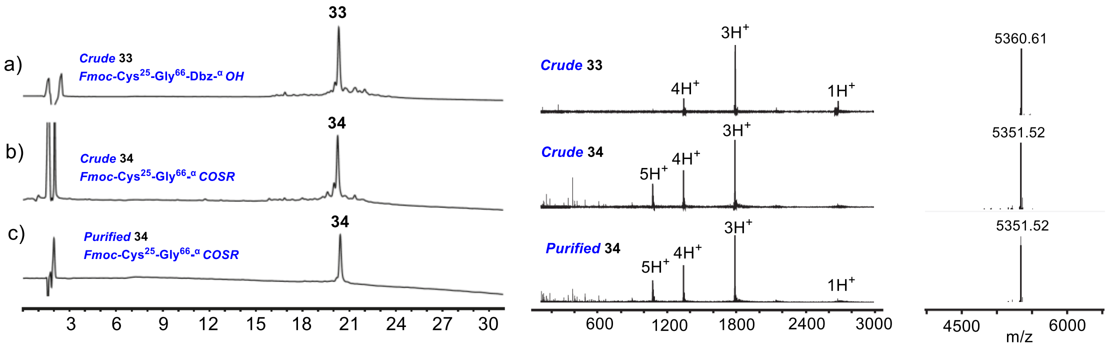

Figure S28. Analytical RP-HPLC profile (λ = 214 nm) together with ESI-MS data (right) for the synthesis of peptide *Fmoc*-Cys^{25}-Gly^{66}- $^{\alpha}$*COSR* (**34**). (a) Chromatogram for the crude peptide *Fmoc*-Cys^{25}-Gly^{66}-Dbz-$^{\alpha}$*OH* (**33**). (b) Chromatogram for the crude MESNa exchanged product *Fmoc*-Cys^{25}-Gly^{66}- $^{\alpha}$*COSR* (**34**). (c) Chromatogram for the purified MESNa exchanged product *Fmoc*-Cys^{25}-Gly^{66}- $^{\alpha}$*COSR* (**34**). *R* = *–CH$_2$CH$_2$SO$_3$Na.* Linear gradient 10%-64% of B over 27 min including 4 min equilibration using Agilent Zorbax SB-C3, 5µm, 4.6 x 150mm, 120Å LC column with 0.9 mL/min flow rate was used for all the chromatographic separation. Purification of the peptide was performed using a linear gradient of 25%-55% buffer B in buffer A over 60 min with a flow rate of 5 mL/min at 40 °C (buffer A = 0.1% TFA in water; buffer B = 0.08% TFA in acetonitrile) using a C3, 9.4 x 250 mm column (Agilent zorbax, 300 Å, 5 µm).

9.3. Synthesis of barstar A segment Cys^{67}-Ser^{89}- $^{\alpha}$*COOH* (35)

The peptide with the sequence Cys-Glu-Ser-Val-Leu-Gln-Val-Phe-Arg-Glu-Ala-Lys-Ala-Glu-Gly-Ala-Asp-Ile-Thr-Ile-Ile-Leu-Ser-$^{\alpha}$*COOH* (**35**) was obtained by machine-assisted SPPS at elevated temperature (see Section 1.3 for the peptide synthesis protocol). Stepwise synthesis of the peptide was carried out on 2-CTC-resin (substitution = 0.4 mmol/g) with a 0.1 mmol scale. Global deprotection and cleavage of the peptide from the resin using a TFA cocktail followed by HPLC purification gave 30 mg (11.42 µmol, 11.4% yield) of the desired peptide Cys^{67}-Ser^{89}-$^{\alpha}$*COOH* (**35**) (**Figure S29**). Observed mass (ESI-MS): 2492.33 Da (deconvoluted most abundant isotopologue); calculated mass: 2492.30 Da (most abundant isotopologue).

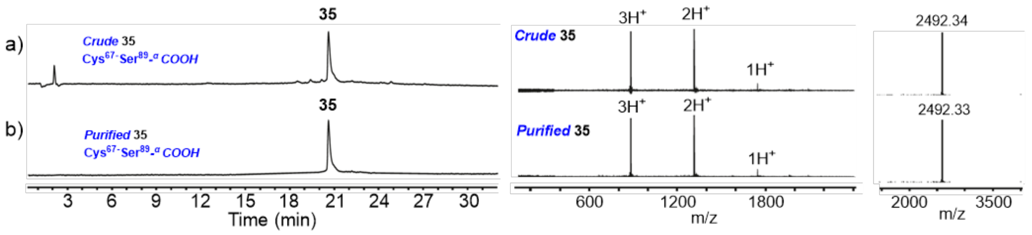

Figure S29. Analytical RP-HPLC profile (λ = 214 nm) together with ESI-MS data (right) for the synthesis of peptide Cys^{67}-Ser^{89}-$^{\alpha}$*COOH* (**35**). (a) Chromatogram for the crude peptide Cys^{67}-Ser^{89}-$^{\alpha}$*COOH* (**35**). (b) Chromatogram for the purified peptide Cys^{67}-Ser^{89}-$^{\alpha}$*COOH* (**35**). Linear gradient 10%-64% of B over 27 min including 4 min equilibration using Agilent Zorbax SB-C3, 5µm, 4.6 x 150mm, 300Å LC column with 0.9 mL/min flow rate was used for all the chromatographic separation. Purification of the peptide was performed using a linear gradient of 20%-55% buffer B in buffer A over 70 min with a flow rate of 5 mL/min at 40 °C (buffer A = 0.1% TFA in water; buffer B = 0.08% TFA in acetonitrile) using a C3, 9.4 x 250 mm column (Agilent zorbax, 300 Å, 5 µm).

9.4. One-pot three-segment ligation of barstar A peptide segments 32, 34, and 35 followed by one-pot desulfurization

To a solution of peptide **34** (5 mg, 0.93 µmol) in 190 µL of the ligation buffer (200 mM PB, 6 M Gdn.HCl, 50 mM TCEP) containing 50 mM MPAA (1.40 mg, 8.32 µmol), was added peptide **35** (2.76 mg, 1.11 µmol) (*Note: Due to the low solubility of peptide **35**, the reaction mixture was slightly gel-like*) and the pH of the reaction mixture was adjusted to 6.7. The first ligation was completed in 6 h to yield the ligated product **34'**. Then, piperidine was added to the reaction mixture, and the pH was adjusted to 11.0 by adding concentrated HCl maintaining the final concentration of piperidine to 20% (v/v) to deprotect the Fmoc group from the ligated product **34'** to yield peptide **36**. Immediately after 7 min of incubation, the pH of the reaction mixture was rapidly reduced to 9.0 by adding concentrated HCl. Solid TCEP (2.9 mg, ~50 mM) was then added, and the pH was readjusted to 7.0 *(Note: After Fmoc-removal, the solution became clear)*. The third peptide segment **32** (3.18 mg, 1.11 µmol) was then added to the reaction mixture, and the pH was readjusted to 6.85. The second and final ligation reaction was completed within 15 h to yield polypeptide **37**. To this solution, N-acetylcysteine (4.90 mg, 30.02 µmol, 100 mM for

0.3 mL) was added solid, and the pH of the reaction mixture was measured and adjusted to 6.5. To this solution, bromoacetamide (1.15 mg, 14.20 µmol) dissolved in 20 µL water was added slowly over 2 minutes (trace amount of MPAA was left unreacted). The reaction mixture was stirred for an extra 2-3 minutes at room temperature. To this solution, TCEP (14.2 mg, 49.54 µmol, 150 mM for 0.33 mL), and VA-044 (10.7 mg, 33.12 µmol, 100 mM for 0.33 mL) were added and the pH of the solution was adjusted to 7.0 and the reaction mixture was kept at 37°C until the completion of the reaction. The desulfurization was completed within 8 h furnishing the full-length Barstar A polypeptide **38**. The one-pot ligations and desulfurization were monitored by analytical HPLC and ESI-MS (main text **Figure 5-C, D,** and **Figure S30**). The purification of the full-length polypeptide by preparative HPLC afforded 3.34 mg (0.33 µmol, 35.48% yield based on the starting peptide segment **34**) of the pure desired peptide **38** (Observed mass (**ESI-MS**): 10147.56 ± 0.14 Da (average of the five most abundant charge states); calculated mass: 10147.47 Da (average isotopic composition)).

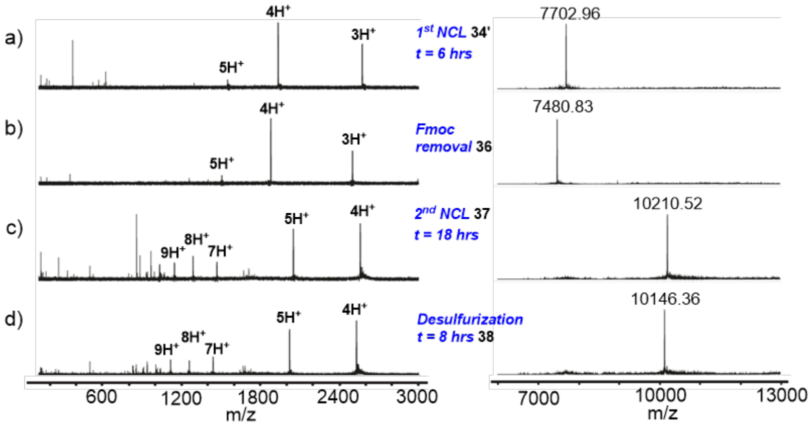

Figure S30. ESI-MS (deconvoluted mass on right) data of the one-pot ligation reactions followed by quenching of MPAA and one-pot desulfurization. ESI-MS data of the (a) ligated polypeptide **34'** after the ligation was over in 6 h (b) Fmoc deprotection from ligated product **34'** to get peptide Cys[25]-Ser[89]-[α]*COOH* (**36**). polypeptide **37** after 2 min of the addition of bromoacetamide (c) second ligation was essentially completed within 18 h to yield ligated product **37** (d) desulfurized polypeptide **38** after quenching MPAA with bromoacetamide and subsequent desulfurization for 8 hrs. RP-HPLC chromatogram data is shown in the main text *Figure 5-C*. Linear gradient 20%-80% of B over 20 min including 4 min equilibration using Agilent Zorbax SB-C3 5 µm 4.6 x 150 mm, 120Å LC column with 0.9 mL/min flow rate was used for all the chromatographic separation. Purification was performed using a linear gradient 25%-60% buffer B in buffer A over 70 min with a flow rate of 5 mL/min at 40 °C (buffer A = 0.1% TFA in water; buffer B = 0.08% TFA in acetonitrile) using a C3, 9.4 x 250 mm column (Agilent zorbax, 300 Å, 5 µm).

9.5. Characterization of barstar A protein (38) by circular dichroism

The chemically synthesized full-length Barstar A polypeptide **38** was characterized by circular dichroism (CD) as mentioned in **section 1.5**. The sample for CD was prepared by initially dissolving lyophilized polypeptide 4 (0.2 mg) in 50 µL of 6 M Gdn.HCl. To this solution, 950 µL buffer (0.02 M PB, 0.1 M NaCl, pH 8.0) was added and kept at 4°C for 30 minutes. To remove the excess Gu.HCl, this solution was dialyzed twice against the above-mentioned buffer at 4°C for 4 h each. Precipitates were observed and removed by centrifugation at 6000 rpm, and the final concentration of the chemically synthesized Barstar A was monitored (0.06 mg/mL). This solution was used for CD spectra measurements and the spectra is shown in **Figure 6-E (main-text)**.

9.6. Inhibition of ribonuclease activity of barnase protein with enhancement of barstar A (38) concentration

Here, we study the inhibition of ribonuclease activity of chemically synthesized Barnase (400 nM) with an increase in the concentration of chemically synthesized Barstar A, a natural inhibitor of Barnase, in the buffer B (0.02 M Tris, 0.1 M NaCl) at pH 8.0 following the reported protocol.[6] The RNA (1 mg/mL) and EtBr (0.077 mM) solutions (1 mL each) were prepared, mixed, and incubated for 30 min. 400 µL volume of this solution was taken and added to the 5.4 mL of buffer B and incubated for 100 s to prepare the stock solution. This stock solution was used for kinetic studies. In the 96-well microplates (Molecular Probes® 96-well microplates), 200 µL of RNA-EtBr stock solution were taken and the fluorescence intensity was monitored for 100 s in the plate reader (POLARstar® Omega). To these solutions, chemically synthesized Barnase (400 nM) pre-incubated together with varying concentrations of Bastar A (0.4 µM, 1 µM, 2.5 µM, and 4

μM) for 10 minutes, along with a control sample without Barstar A, were added and the change in the fluorescence intensity was monitored for next 19 minutes using an excitation and emission wavelength of 520 and 620 nm. To ensure the reliability of the results, the experiment was carried out in triplicate and repeated three times to ensure reproducibility and plotted as an average as shown in **Figure 6-F (main-text)**.

References

[1] J. M. Collins, S. K. Singh, *Coupling Method for Peptide Synthesis at Elevated Temperatures*, **2019**, US10308677B2.

[2] J.-S. Zheng, S. Tang, Y.-K. Qi, Z.-P. Wang, L. Liu, *Nat Protoc* **2013**, *8*, 2483–2495.

[3] D. Han, Y. Cui, X. Deng, C. Li, X. Zhu, B. Wang, G.-C. Chu, Z. A. Wang, S. Tang, J.-S. Zheng, L.-J. Liang, L. Liu, *J. Am. Chem. Soc.* **2025**, DOI 10.1021/jacs.4c13464.

[4] D. T. Flood, J. C. J. Hintzen, M. J. Bird, P. A. Cistrone, J. S. Chen, P. E. Dawson, *Angew Chem Int Ed Engl* **2018**, *57*, 11634–11639.

[5] A. Kar, J. Mannuthodikayil, S. Singh, A. Biswas, P. Dubey, A. Das, K. Mandal, *Angewandte Chemie International Edition* **2020**, *59*, 14796–14801.

[6] D. R. Tripathy, A. K. Dinda, S. Dasgupta, *Anal Biochem* **2013**, *437*, 126–129.
